# Yin Yang 1 Promotes Antiprogrammed Cell Death‐1 Resistance in Hepatocellular Carcinoma through Polypeptide N‐Acetylgalactosaminyltransferase 16‐Mediated Glycosylation of Programmed Death Ligand‐1

**DOI:** 10.1002/mco2.70504

**Published:** 2025-11-26

**Authors:** Shu‐sheng Lin, Gang Xiao, Qin‐qin Liu, Jia‐hao Xue, Zhi‐jun Chen, Hong‐hua Zhang, Xiang‐ping Zhu, Keng‐long Huang, Cai‐ni Yang, Ke Zhu, Hao‐ming Lin, Rui Zhang

**Affiliations:** ^1^ Department of Biliary‐Pancreatic Surgery Sun Yat‐sen Memorial Hospital Sun Yat‐sen University Guangzhou China; ^2^ Guangzhou Key Laboratory of Precise Diagnosis and Treatment of Biliary Tract Cancer Sun Yat‐sen Memorial Hospital Sun Yat‐sen University Guangzhou China; ^3^ Department of Thoracic Surgery Guangzhou First People's Hospital Guangzhou Medical University Guangzhou China; ^4^ Center For Medical Research On Innovation and Translation Guangzhou First People's Hospital Guangzhou Medical University Guangzhou China; ^5^ Department of Hepatobiliary Surgery Southwest Hospital Third Military Medical University (Army Medical University) Chongqing China; ^6^ Department of Oncology Sun Yat‐Sen Memorial Hospital Sun Yat‐Sen University Guangzhou China

**Keywords:** hepatocellular carcinoma, immune checkpoint inhibitors, polypeptide N‐acetylgalactosaminyltransferase 16, programmed death ligand‐1 glycosylation, Yin Yang 1

## Abstract

Immune checkpoint inhibitors (ICIs) are widely used for treating hepatocellular carcinoma (HCC), yet their efficacy remains limited, with suboptimal response rates. The predictive power of the current biomarker, programmed death ligand‐1 (PD‐L1), is limited by detection variability and glycosylation, underscoring the need for complementary biomarkers to enhance predictive accuracy. In this study, mass spectrometry was employed to identify proteomic alterations in HCC tissues from responders and nonresponders to anti‐programmed cell death‐1 (PD‐1) therapy. Survival analysis established the role of Yin Yang 1 (YY1) in determining ICI efficacy. Coculture models of hepatoma and CD8^+^ T cells revealed the immunosuppressive function of YY1. Transcriptome sequencing identified polypeptide N‐acetylgalactosaminyltransferase 16 (GALNT16) as a transcriptional target of YY1, and subsequent Western blot and coimmunoprecipitation assays demonstrated that GALNT16 augments PD‐L1 expression. Furthermore, in vivo mouse models demonstrated that YY1 knockdown potentiated the efficacy of anti‐PD‐1 therapy, an effect that was partially reversed by GALNT16 overexpression. Specifically, YY1 upregulates GALNT16, which in turn promotes PD‐L1 glycosylation and stability, leading to diminished CD8^+^ T cell activity. Thus, GALNT16 knockdown rescued the compromised CD8^+^ T cell cytotoxicity induced by YY1. Collectively, these results elucidate the YY1/GALNT16/PD‐L1 axis as a pivotal mechanism underlying HCC resistance to ICI therapy. This highlights the therapeutic potential of targeting PD‐L1 glycosylation pathways.

## Graphical abstract

Mass‐spectrometric profiling of tumor tissues from anti‐PD‐1‐responsive versus non‐responsive HCC patients identified the transcription factor YY1 as a key regulator. YY1 transcriptionally upregulates the glycosyltransferase GALNT16, which binds PD‐L1 and catalyzes N219 glycosylation. This posttranslational modification stabilizes PD‐L1 on the cell surface, thereby enhancing its interaction with PD‐1 on CD8^+^ T cells, promoting effector‐cytokine secretion (e.g., IFNγ) and potentiating cytotoxic activity (e.g., upregulation of CD107a) in CD8^+^ T cells, and ultimately impairing the efficacy of anti‐PD‐1 therapy.

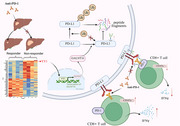

## 1 Introduction

Hepatocellular carcinoma (HCC) is a complex and heterogeneous malignant tumor that poses significant challenges to effective treatment [1]. Among the various therapeutic strategies, immune checkpoint inhibitors (ICIs), particularly those targeting the programmed death ligand‐1 (PD‐L1) and programed cell death‐1 (PD‐1) pathway, have shown great promise in activating the body's immune system to combat tumor growth [2, 3]. PD‐L1, a pivotal molecule in this signaling network, is frequently overexpressed in HCC, and its engagement with PD‐1 on T cells leads to the dampening of T cell‐driven immune responses. Consequently, the surface expression of PD‐L1 on malignant cells is a critical determinant of ICI efficacy and plays a central role in the success of immunotherapeutic interventions [4, 5].

Nonetheless, PD‐L1 expression levels are not always predictive of ICI efficacy. Numerous studies have found that certain PD‐L1‐negative patients can also benefit from anti‐PD‐L1 and anti‐PD‐1 therapy, likely due to a combination of factors such as the heterogeneity of PD‐L1 expression, variations in detection methods, glycosylation modifications of the PD‐L1 molecule, and the dynamic changes in the tumor immune microenvironment [6]. Among these, the glycosylation of PD‐L1 has garnered significant attention in recent years, with research indicating that it is involved in regulating the stability of PD‐L1 and affecting its interaction with PD‐1 and subsequent immune responses [7, 8]. Lee et al. [9] demonstrated that the binding affinity of the monoclonal antibody to cell surface PD‐L1 antigen increased approximately 25‐fold and 55‐fold in A549 and H1299 cells after deglycosylation. In addition, aberrant glycosylation of PD‐L1 diminishes the efficacy of anti‐PD‐1 therapy in cancer cell lines, exhibiting resistance to treatment [10]. The presence of distinct glycan patterns on PD‐L1 can augment its affinity for PD‐1, which may contribute to the development of resistance to ICIs. Our previous study also found that N‐glycosylation in HCC can promote the progression of HCC and exert immunosuppressive effects, affecting the therapeutic effect of ICIs [11]. It is evident that the glycosylation modifications of PD‐L1 impact the clinical application of ICIs in various aspects, including PD‐L1 positivity assessment and sensitivity.

The present study has identified Yin Yang 1 (YY1), a pleiotropic transcription factor, as a key player associated with resistance to ICIs in HCC. There is an accumulating body of evidence indicating that YY1 plays a role in regulating a number of processes associated with cancer progression, including cellular proliferation, differentiation, metabolic reprogramming, and apoptosis [12, 13, 14, 15]. Moreover, emerging evidence indicates that it may also influence tumor immunity. In tumor‐associated macrophages, YY1 induces a phenotypic shift from antitumor M1 macrophages to immunosuppressive M2 macrophages by inhibiting miR‐125a [16]. In tumor cells, YY1 induces the transcription of the inhibitory immune checkpoint FGL1, which results in T cell apoptosis and the formation of an immunosuppressive microenvironment in lung adenocarcinoma [17]. Furthermore, evidence indicates that YY1 plays a role in PD‐L1 upregulation, both through direct transcriptional regulation of PD‐L1 and indirect induction of IL8 expression [18, 19]. Nevertheless, it is not yet clear whether YY1 is involved in the mediation of PD‐L1 glycosylation modifications.

The present study identified the glycosyltransferase GALNT16 as a direct downstream gene of YY1 and elucidated the mechanism through which GALNT16 enhances PD‐L1 glycosylation, thereby increasing its stability. These findings suggest that YY1 may also influence the expression and function of PD‐L1 by promoting its glycosylation. These findings contribute to our understanding of YY1‐mediated tumor immune regulation and present a potential therapeutic target for enhancing the efficacy of ICIs in HCC by targeting the glycosylation modifications of PD‐L1.

## 2 Results

### 2.1 Proteomic Analysis Uncovers the Critical Role of YY1 in HCC Immunotherapy

**FIGURE 1:**
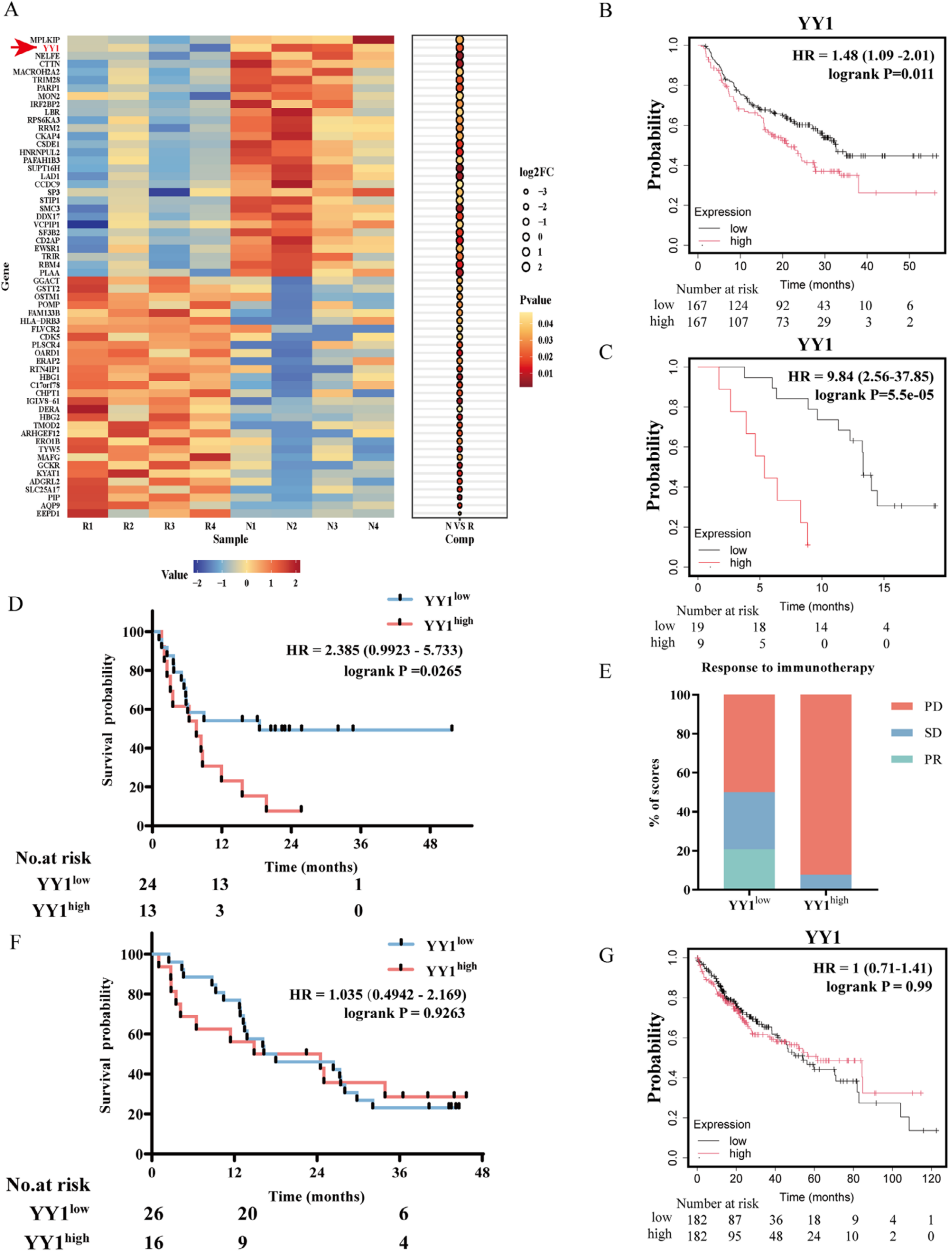
Proteomic profiling and clinical validation identify YY1 as a predictive biomarker for anti‐PD‐1 therapy response in HCC. (A) Heatmap displaying the top 30 differentially expressed proteins (DEPs) between anti‐PD‐1 therapy responders and nonresponders in HCC tissues, identified by proteomic analysis. (B) Kaplan–Meier analysis of overall survival (OS) in anti‐PD‐1‐treated melanoma patients (KM‐Plotter database cohort, n = 334), stratified by high versus low YY1 expression. (C) Kaplan–Meier analysis of OS in anti‐PD‐1‐treated glioblastoma patients (KM‐Plotter database cohort, n = 28), grouped by YY1 expression levels. (D) Progression‐free survival (PFS) analysis of anti‐PD‐1‐treated HCC patients in our institutional cohort (n = 37), categorized by YY1 expression. (E) Comparative analysis of immunotherapy response categories (PD, SD, PR) in HCC patients (n = 37) stratified by YY1 expression levels. Abbreviations: PD, progressive disease; SD, stable disease; PR, partial response. (F) PFS analysis of HCC patients receiving non ‐immunotherapy treatments in our cohort (n = 42), grouped by YY1 expression. (G) OS analysis of HCC patients from the KM‐Plotter database cohort (*n* = 370), stratified by YY1 expression.

To identify differentially expressed proteins (DEPs) among HCC patients with varying responses to anti‐PD‐1 therapy, we conducted a proteomic analysis using mass spectrometry on tissue samples from patients receiving anti‐PD‐1 therapy. This scrutiny identified 139 DEPs, from which we meticulously selected the top 30 upregulated and top 30 downregulated proteins based on their fold change across treatment‐resistant and responsive groups, as illustrated in the heatmap (Figure 1A). Notably, among these DEPs, mitotic PLK1 interacting protein (MPLKIP) exhibited the largest fold change, followed immediately by the transcription factor YY1 (Figure 1A). We next asked whether either protein could reliably predict anti‐PD‐1 therapeutic efficacy. For this purpose, the Kaplan–Meier Plotter database cohort was analyzed using overall survival (OS) as the endpoint, whereas our own cohort was evaluated using progression‐free survival (PFS) as the primary endpoint. MPLKIP stratified OS only in glioblastoma (Figure S1A), whereas its prognostic impact did not reach statistical significance in melanoma (Figure S1B) or in our 37‐patient HCC cohort (Figure S1C). In contrast, YY1—an established regulator of tumor immunomodulation—exhibited consistent predictive power: elevated YY1 expression was associated with shorter OS in melanoma (Figure 1B) and glioblastoma (Figure 1C) patients receiving anti‐PD‐1 therapy. Extending these observations to HCC, we profiled YY1 in the same 37 patients (clinical characteristics in Table S1) treated with anti‐PD‐1 blockade and confirmed that high YY1 expression independently predicted markedly shortened PFS (Figure 1D). Consistently, within this cohort, patients with high YY1 expression exhibited a higher incidence of progressive disease, as assessed by immune‐related response evaluation criteria in solid tumors (iRECIST) (92.3% vs. 50%; *p* = 0.0129) (Figure 1E).

To determine whether YY1‐associated progression is immunotherapy specific, we analyzed an independent cohort of 42 HCC patients managed with conventional, nonimmunotherapy regimens (clinical characteristics in Table S2). In this setting, YY1 expression did not stratify PFS (Figure 1F), nor did it influence OS in the Kaplan–Meier Plotter dataset (Figure 1G). Collectively, these data indicate that the prognostic relevance of YY1 in HCC is most pronounced in the context of anti‐PD‐1 therapy, whereas its predictive value is markedly attenuated under nonimmunotherapy regimens.

### 2.2 YY1 Expression Exhibits a Negative Correlation with CD8^+^ T Cell Infiltration and Response to Immunotherapy

**FIGURE 2:**
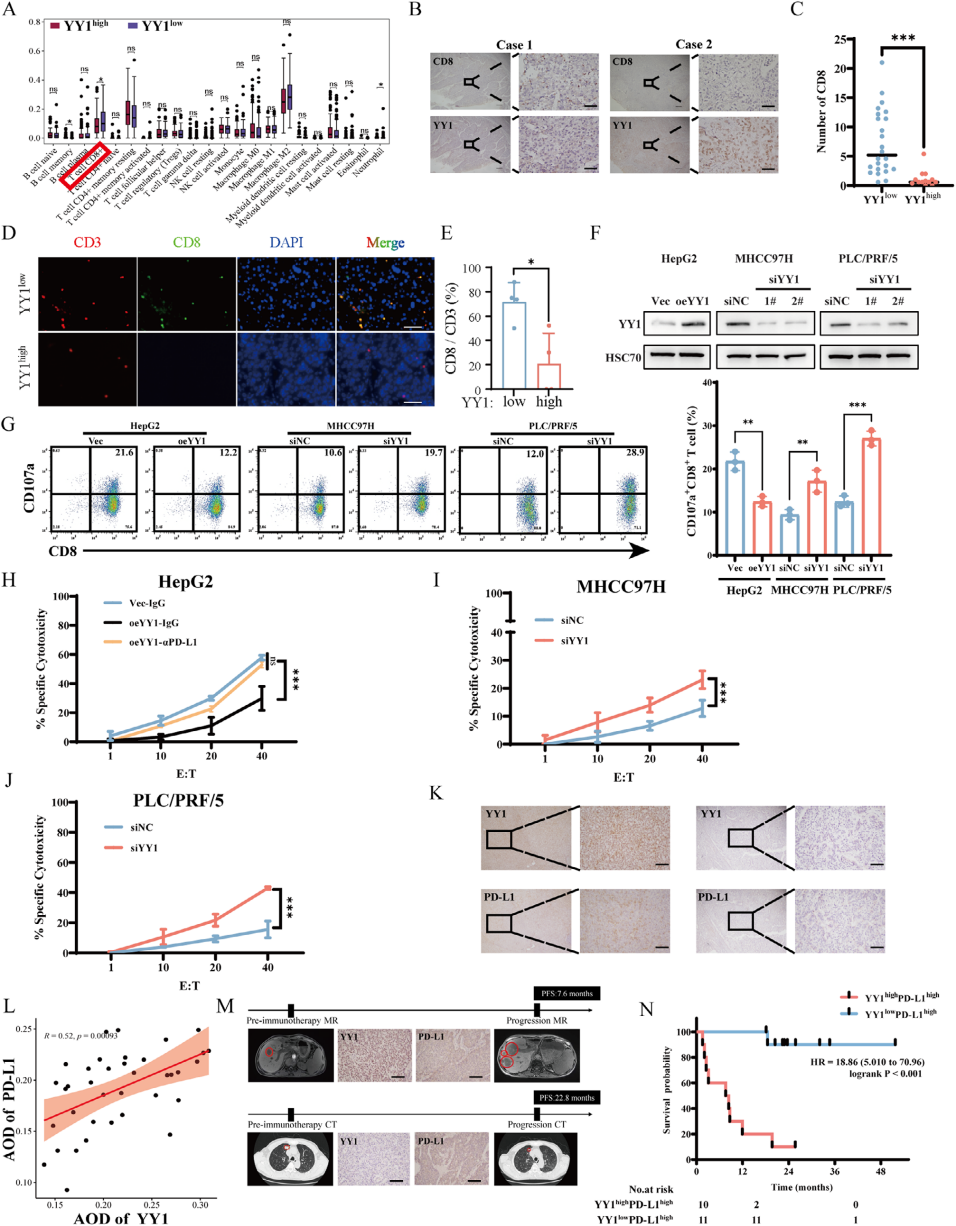
YY1 expression correlates with impaired cytotoxicity and infiltration of CD8+ T cell and anti‐PD‐1 resistance in HCC. (A) Differential immune cell infiltration patterns between YY1‐high and YY1‐low HCC tumors from the TCGA‐LIHC cohort, were analyzed using CIBERSORT deconvolution algorithm. Bar heights represent mean proportions of 22 immune cell subtypes. (B) Representative immunohistochemical staining of consecutive sections showing inverse spatial correlation between YY1 (brown nuclear) and CD8^+^ T cells (brown membranous/cytoplasmic). Scale bars: 200 µm (overview), 50 µm (inset). (C) Quantification of CD8^+^ T cell density (cells/field) in YY1‐high vs. YY1‐low groups (*n* = 37, unpaired *t*‐test, ***p < 0.001). Five random 400× fields per sample analyzed. (D) Multiplex immunofluorescence showing CD3^+^ (red), CD8^+^ (green), and DAPI (blue) in representative YY1‐high/low tumors. Scale bar: 50 µm. (E) CD8^+^/CD3^+^ ratio quantification (*n* = 4/group, unpaired *t*‐test). (F) Western blot validation of YY1 manipulation in HCC cell lines: overexpression in HepG2 (oeYY1) vs. vector control; knockdown in MHCC97H and PLC/PRF/5 (siYY1) vs. siNC controls. HSC70 served as loading control. (G) Flow cytometric analysis of CD107a^+^ cytotoxic CD8^+^ T cells after 24 h coculture with modified HCC cells (E:T = 10:1). Right panel shows the quantified CD107a^+^CD8^+^ percentages (*n* = 3 experiments, paired *t*‐test, **p < 0.01, ***p < 0.001). (H–J) LDH release assays quantifying tumor cell lysis by activated CD8^+^ T cells across E:T ratios. (H) PD‐L1 blockade effects in YY1‐overexpressing HepG2; (I and J) YY1 knockdown effects in MHCC97H, PLC/PRF/5. % Specific cytotoxicity calculated as: [(experimental − effector spontaneous − target spontaneous)/(target maximum − target spontaneous)] × 100. Data shown as mean ± SD (*n* = 3). (K) Consecutive section IHC demonstrating spatial overlapping expression patterns of YY1 and PD‐L1 in representative HCC cases. Scale bar: 100 µm. Scale bar: 100 µm. (L) Positive correlation between YY1 and PD‐L1 expression (*n* = 37, Pearson's *r* = 0.52, *p* = 0.00093). The average optical density (AOD) of YY1 and PD‐L1 staining quantified using ImageJ. (M) Radiographic‐CT/MRI and paired IHC images showing differential anti‐PD‐1 responses in PD‐L1^+^ patients stratified by YY1 levels. Tumor masses circled in red. Scale bar: 100 µm. (N) Progression‐free survival analysis in PD‐L1^high^ HCC patients receiving anti‐PD‐1 therapy (YY1‐high vs. low, log‐rank *p* < 0.0001).

For a more profound comprehension of YY1's role in immunotherapy outcomes, we engaged bioinformatics analysis to explore the link between YY1 expression and immune cell infiltration within the tumor microenvironment. Using the CIBERSORT deconvolution algorithm on RNA‐seq profiles of primary HCC tumors from the Cancer Genome Atlas Liver Hepatocellular Carcinoma (TCGA‐LIHC) cohort, we found that YY1‐low tumors exhibited a significantly higher proportion of CD8⁺ cytotoxic T cells than YY1‐high tumors (Figure 2A). To reinforce this finding, we utilized immunohistochemistry (IHC) and multiplex immunofluorescence (mIF) to confirm the negative correlation between YY1 expression and CD8^+^ T cell infiltration in HCC tissues (Figure 2B–E). Additionally, in vitro coculture assays revealed that elevated YY1 in hepatoma cells markedly suppressed CD8^+^ T‐cell activation, whereas YY1 knockdown augmented tumor‐inhibitory capacity, evidenced by increased CD107a surface exposure—a surrogate marker of cytotoxic granule release (Figure 2F–I, J). Upon target‐cell recognition, CD8^+^ T cells externalize CD107a during perforin/granzyme exocytosis, rendering CD107a a reliable read‐out of cytotoxic activity [20, 21]. Collectively, these results suggest that the modulation of YY1 expression in tumor cells could significantly regulate CD8^+^ T cell activity.

Given the significance of PD‐L1 expression on tumor cells in modulating CD8^+^ T cell function and the effectiveness of anti‐PD‐L1 and anti‐PD‐1 therapy, we proceeded to examine the correlation between PD‐L1 and YY1 expression more closely. Our analysis illustrated a positive association between PD‐L1 and YY1 expression in HCC (Figure 2K, L). Although higher PD‐L1 expression is typically considered favorable for anti‐PD‐L1and anti‐PD‐1 treatment responses, our review of clinical imaging reports from HCC patients treated with anti‐PD‐1 revealed that high PD‐L1 expression does not consistently predict strong immune responses. Intriguingly, this correlation appears to be closely tied to the levels of YY1 expression. Notably, patients with lower YY1 expression seem to experience greater benefits from immunotherapy (Figure 2M). Moreover, the survival analysis outcomes corroborate this assertion (Figure 2N).

### 2.3 The Transcription Factor YY1 Enhances the Stability of PD‐L1 Protein

**FIGURE 3:**
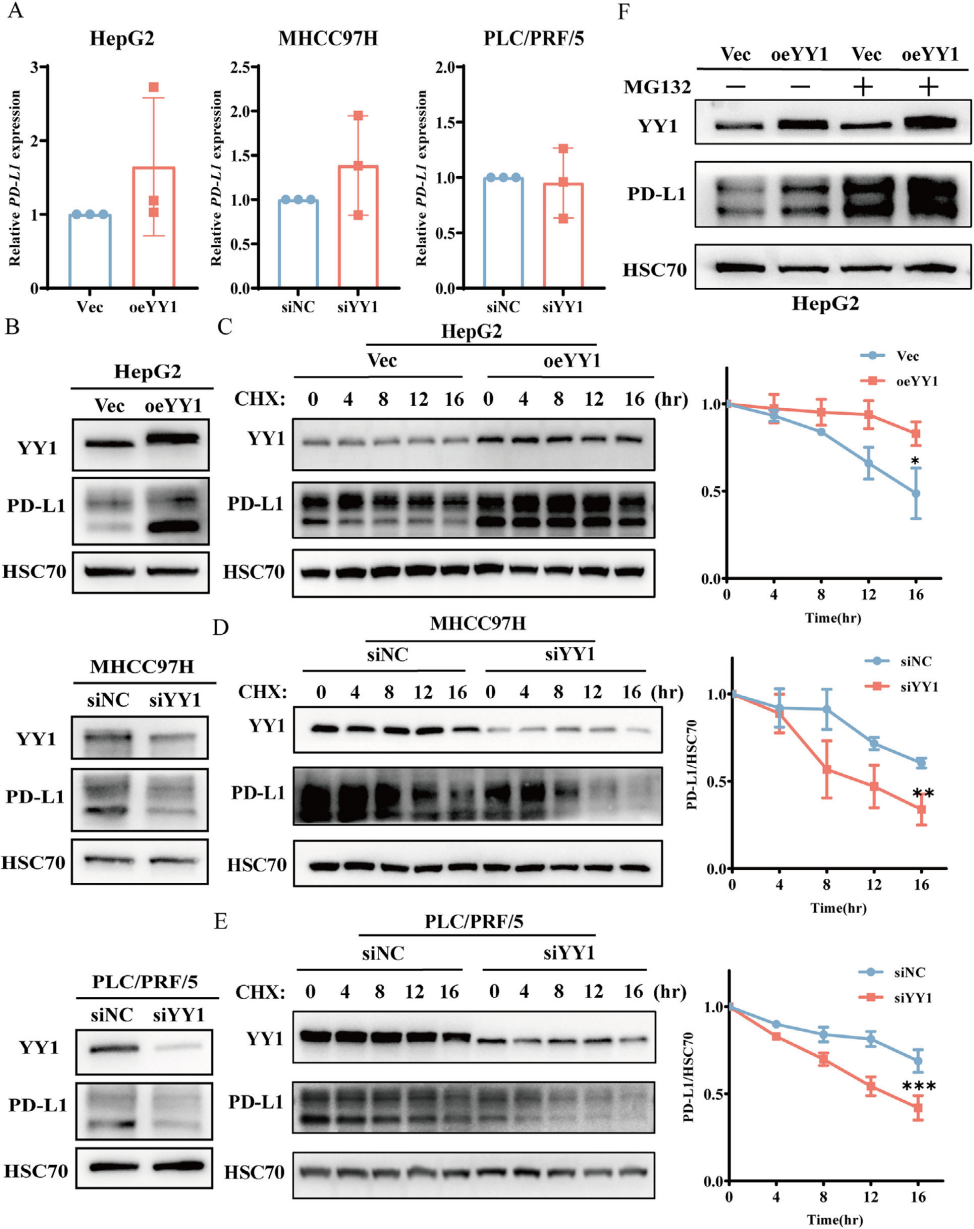
YY1 stabilizes PD‐L1 protein by inhibiting proteasomal degradation in hepatocellular carcinoma cells. (A) RT‐qPCR quantification of *CD274* (PD‐L1) mRNA levels in HepG2 (vector vs. oeYY1) and in MHCC97H and PLC/PRF/5 cells (siNC vs. siYY1). mRNA levels normalized to *ACTB* (β‐actin) using the 2^−ΔΔCt^ method (*n* = 3 experiments, mean ± SD; ns: not significant by unpaired *t*‐test). (B) Western blot validation of YY1 manipulation efficiency and corresponding PD‐L1 protein expression. HSC70 served as loading control. HepG2: vector vs. oeYY1; MHCC97H and PLC/PRF/5: siNC vs. siYY1 (representative of three replicates). (C–E) Cycloheximide (CHX, 50 µg/mL) chase assays. PD‐L1‐overexpressing cells were transfected as indicated (vector or oeYY1 in HepG2; siNC or siYY1 in MHCC97H and PLC/PRF/5), then harvested at 0, 4, 8, 12, or 16 h post‐CHX. Western blots (left) and quantified PD‐L1/HSC70 decay curves (right) demonstrate YY1‐mediated PD‐L1 stabilization (*n* = 3; two‐way ANOVA with Tukey's posthoc test). *p < 0.05, **p < 0.01, ***p < 0.001. (F) HepG2 cells (vector or oeYY1) were treated with/without MG132 (10 µM, 6 h) and immunoblotted for YY1 and PD‐L1; HSC70 was the loading control (*n* = 3).

Given the observed positive correlation between the transcription factor YY1 and the expression levels of PD‐L1, we initially investigated the possibility that YY1 may transcriptionally regulate PD‐L1. As evidenced by quantitative real‐time PCR (RT‐qPCR), modulation of YY1 expression did not alter the transcriptional levels of PD‐L1 (Figure 3A). In contrast, Western blot (WB) analysis revealed that YY1 could upregulate the expression of PD‐L1 protein (Figure 3B). This suggests that YY1 may modulate PD‐L1 expression at the translational or posttranslational level. To test this, hepatoma cells were treated with the protein synthesis inhibitor cycloheximide (CHX) to assess the effect of YY1 on PD‐L1 protein stability. The results showed that overexpression of YY1 enhanced PD‐L1 protein stability (Figure 3C), while YY1 knockdown significantly reduced it (Figure 3D, E). Furthermore, the upregulatory effect of YY1 on PD‐L1 protein levels was abrogated by treatment with the proteasome inhibitor MG132 (Figure 3F). Collectively, these findings indicate that YY1 promotes PD‐L1 protein levels by inhibiting ubiquitin–proteasome‐mediated degradation of PD‐L1.

### 2.4 YY1 Regulates the Stability of PD‐L1 Through GALNT16‐Mediated Glycosylation Modification

**FIGURE 4:**
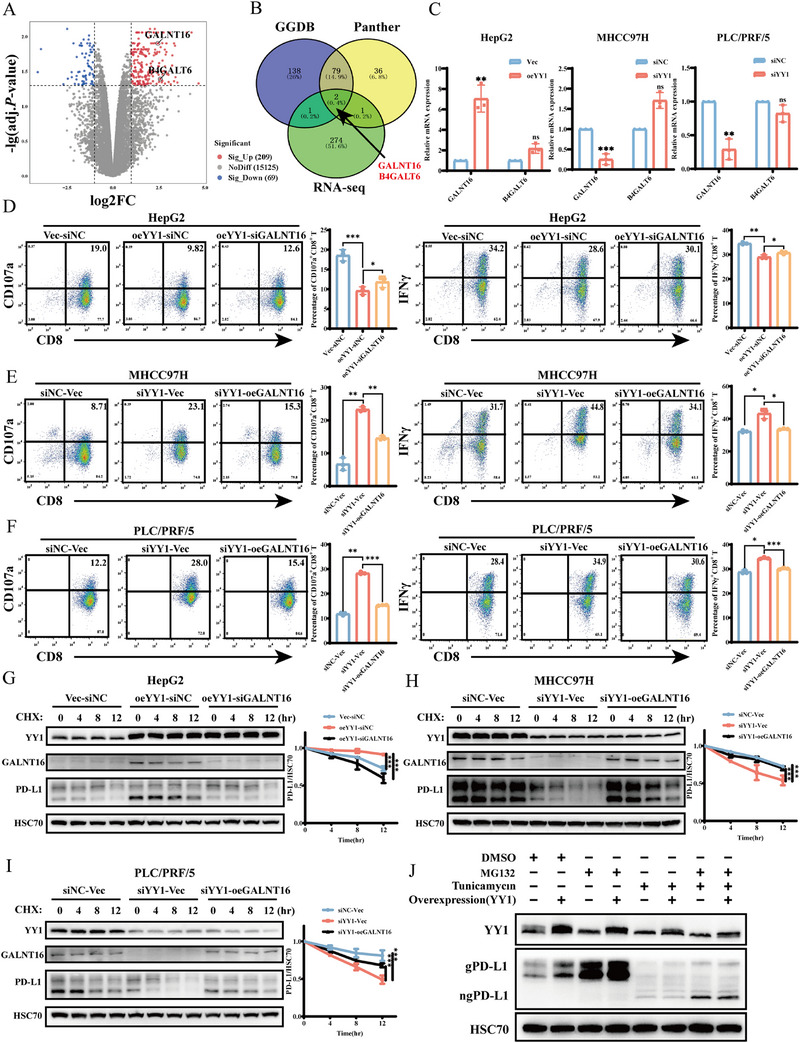
YY1 regulates PD‐L1 stability via GALNT16‐mediated glycosylation. (A) Volcano plot of bulk RNA‐seq data (*n* = 3 biological replicates per group) comparing HepG2 cells overexpressing YY1 versus vector control. Differentially expressed genes (DEGs) were identified with the limma package (R); cut‐off: |log_2_FC| ≥ 1 and adjusted *p* < 0.05. Upregulated genes are shown in red, downregulated genes in blue, and nonsignificant genes in gray. (B) Venn diagram depicting the overlap between the complete DEG set (up‐ and downregulated) and glycosylation‐related gene lists curated in the GGDB and PANTHER databases. (C) RT‐qPCR validation of GALNT16 and B4GALT6 mRNA levels in HepG2 (oeYY1 vs. vector), MHCC97H (siYY1 vs. siNC) and PLC/PRF/5 (siYY1 vs. siNC) cells. Data were normalized to ACTB (2^–ΔΔCt^) and are presented as mean ± SD of three independent experiments; ns, not significant; **p < 0.01, ***p < 0.001 by unpaired *t*‐test. (D–F) Flow cytometric quantification of cytotoxic (CD107a⁺) and activated (IFNγ⁺) CD8⁺ T cells after 24 h coculture (E:T = 10:1) with: (D) HepG2 cells (vector, oeYY1, oeYY1 + siGALNT16); (E) MHCC97H cells (siNC, siYY1, siYY1 + oeGALNT16); (F) PLC/PRF/5 cells (siNC, siYY1, siYY1 + oeGALNT16). Bar graphs to the right of each dot‐plot summarize the percentages of CD107a⁺ or IFNγ⁺ CD8⁺ T cells (mean ± SD, *n* = 3; paired *t*‐test; *p < 0.05, **p < 0.01, ***p < 0.001). (G) Cycloheximide (CHX, 50 µM) chase assay in PD‐L1‐expressing HepG2 cells treated as vector + siNC, oeYY1 + siNC, or oeYY1 + siGALNT16. Representative Western blots (left) show YY1, GALNT16, and PD‐L1 with HSC70 as loading control. The right panel presents the corresponding PD‐L1 degradation curve normalized to HSC70 (mean ± SD, *n* = 3; two‐way ANOVA with Tukey's posthoc test; *p < 0.05, **p < 0.01, ***p < 0.001). (H and I) CHX chase (50 µM) in (H) PD‐L1‐expressing MHCC97H and (I) PD‐L1‐expressing PLC/PRF/5 cells treated as siNC + vector, siYY1 + vector, or siYY1 + oeGALNT16. Representative Western blots (left) and the PD‐L1 degradation curves normalized to HSC70 (right) are shown (mean ± SD, *n* = 3; two‐way ANOVA with Tukey's posthoc test; *p < 0.05, **p < 0.01, ***p < 0.001). (J) Western blot of HepG2 cells (vector, oeYY1) treated sequentially with ±tunicamycin (2 µg/mL, 18 h) and ±MG132 (10 µM, 6 h). HSC70 served as loading control. gPD‐L1 denotes glycosylated PD‐L1; ngPD‐L1 denotes nonglycosylated PD‐L1.

Considering that the combined expression of PD‐L1 and YY1 has a better predictive value for immune therapy outcomes than PD‐L1 expression alone, we hypothesized that YY1's regulation of PD‐L1 might involve other posttranslational modifications beyond ubiquitination. Recent studies have indicated that glycosylation is a major posttranslational modification affecting PD‐L1 activity. Therefore, we screened for glycosylation‐related genes potentially influenced by YY1 through transcriptome sequencing (Figure 4A). By comparing the differentially expressed genes from sequencing results with the glycosylation gene sets in the GlycoGene Database (GGDB) and PANTHER databases, we identified GALNT16 and B4GALT6 as potential downstream target genes of YY1 (Figure 4B). RT‐qPCR experiments confirmed the regulatory relationship of YY1 on GALNT16 (Figure 4C). In the tumor cell‐CD8⁺ T cell coculture system, genetic rescue experiments demonstrated functional restoration of CD8⁺ T cell activity: knocking down GALNT16 in YY1‐overexpressing tumor cells (Figure 4D) or overexpressing GALNT16 in YY1‐knockdown cells (Figure 4E, F) both reversed YY1‐mediated CD8⁺ T cell impairment. Flow cytometric quantification of CD107a surface exposure—a direct indicator of degranulation‐mediated cytotoxic activity [20, 21]—and intracellular IFNγ production, a hallmark of effector cytokine secretion and T cell activation [22, 23], showed that YY1 predominantly suppresses CD8^+^ T cell function through a GALNT16‐dependent pathway.

Furthermore, we found that there is no difference in the mRNA levels of PD‐L1 (*CD274*), regardless of whether GALNT16 is overexpressed or silenced (Figure S2A). Subsequently, we explored whether GALNT16 mediates the regulatory effect of YY1 on PD‐L1. Knocking down GALNT16 in cells overexpressing YY1 significantly inhibited PD‐L1 expression and reduced its glycosylation modification level (Figure 4G). Conversely, overexpressing GALNT16 in YY1‐knockdown cells increased glycosylation of PD‐L1 protein (Figures 4H, I and S2B). This suggests that YY1 might promote the glycosylation of PD‐L1 by upregulating GALNT16. Given that glycosylation of PD‐L1 can enhance its stability, we further investigated whether YY1‐mediated glycosylation of PD‐L1 affects its protein expression. We treated YY1‐overexpressing hepatoma cells or control cells with the proteasome inhibitor MG132 and the glycosylation inhibitor tunicamycin alone or in combination (Figure 4J). The results showed that nonglycosylated PD‐L1 (ngPD‐L1) was barely visible in cells without inhibitor treatment, while PD‐L1 induced by YY1 was all in glycosylated form (gPD‐L1). Treatment with tunicamycin alone significantly inhibited PD‐L1 expression in tumor cells, and this inhibitory effect was independent of YY1 expression levels, indicating that glycosylation indeed enhanced the stability of PD‐L1. Although MG132 alone showed higher levels of gPD‐L1 in YY1‐overexpressing cells, the combination treatment with tunicamycin resulted in no difference in either gPD‐L1 or ngPD‐L1 between YY1‐overexpressing or control cells, confirming that the regulation of PD‐L1 protein levels by YY1 is related to glycosylation modification and supporting our hypothesis that YY1 does not affect PD‐L1 expression at the translational level.

### 2.5 GALNT16 May Inhibit the Ubiquitination Degradation of PD‐L1 by Promoting Its Glycosylation

**FIGURE 5:**
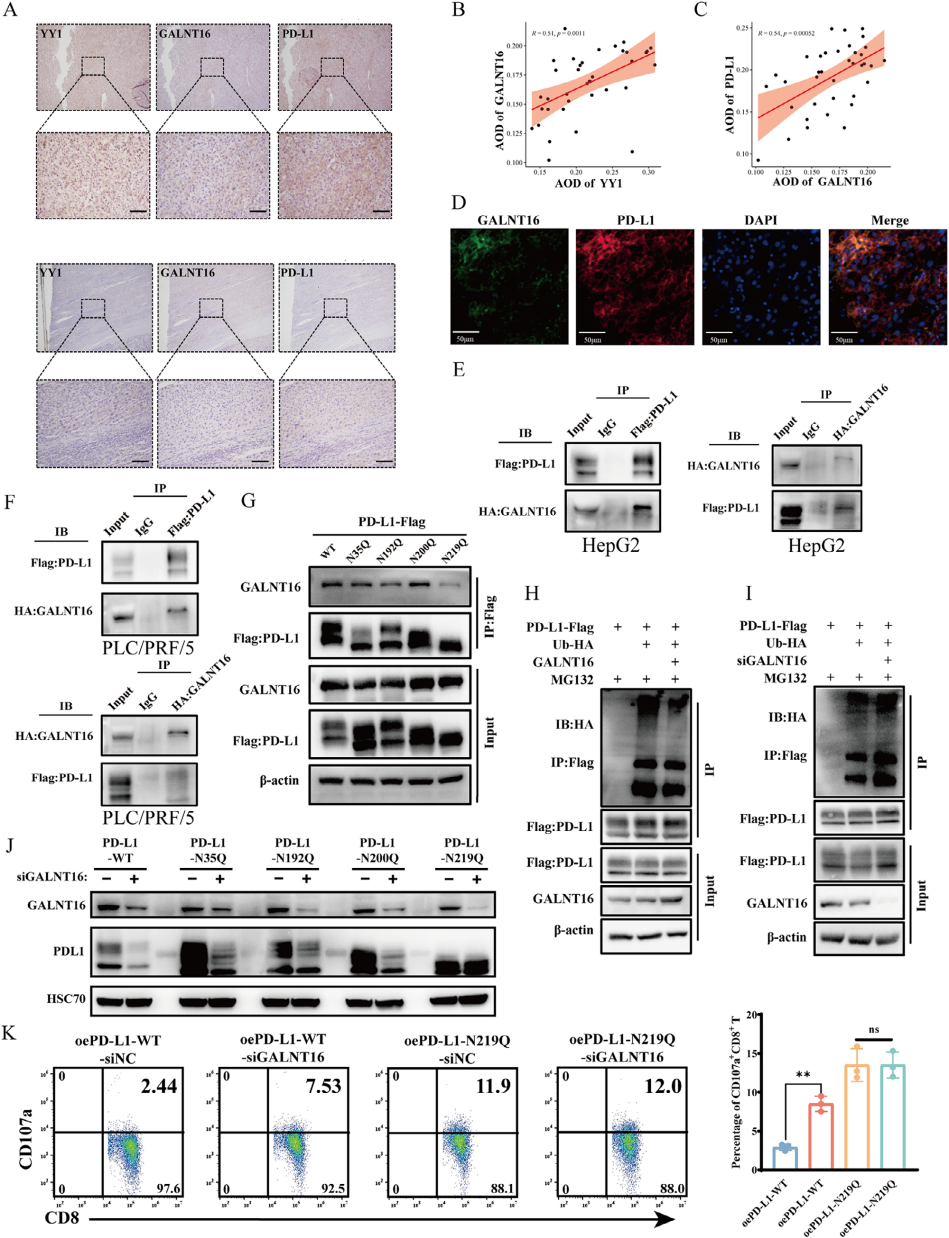
GALNT16 impairs PD‐L1 ubiquitination by enhancing its glycosylation. (A) Representative immunohistochemical staining of consecutive human HCC sections showing expression of YY1, GALNT16, and PD‐L1. Scale bar, 100 µm. (B) Correlation between YY1 and GALNT16 average optical density (AOD) in 37 anti‐PD‐1–treated HCC tissues (Pearson's r = 0.51, p = 0.0011). AOD was quantified with ImageJ. (C) Correlation between GALNT16 and PD‐L1 AOD in the same cohort (Pearson's r = 0.54, p = 0.0005). (D) Multiplex immunofluorescence of HCC sections demonstrating colocalization of GALNT16 (green) and PD‐L1 (red); nuclei counterstained with DAPI (blue). Scale bar, 50 µm. (E and F) Coimmunoprecipitation in HepG2 (E) and PLC/PRF/5 (F) cells overexpressing Flag‐PD‐L1 and HA‐GALNT16. Left: anti‐Flag immunoprecipitates immunoblotted for HA (GALNT16); right: anti‐HA immunoprecipitates immunoblotted for Flag (PD‐L1). Data are representative of three independent experiments. (G) Co‐IP from HepG2 cells transfected with Flag‐tagged wild‐type or glycosylation‐site mutants (N35Q, N192Q, N200Q, N219Q) of PD‐L1 and immunoblotted for endogenous GALNT16. Representative of *n* = 3. (H and I) Ubiquitination assay. HepG2 cells expressing Flag‐PD‐L1 ± HA‐Ub were treated with MG132 (10 µM, 6 h). Co‐IP shows PD‐L1 ubiquitination upon GALNT16 overexpression (H) or knockdown (I). Representative of *n* = 3. (J) Western blot of Flag‐PD‐L1 (WT or indicated mutants) in HepG2 cells with or without GALNT16 knockdown. HSC70 served as loading control. Representative of *n* = 3. (K) Flow cytometric analysis of CD8⁺ T cells cocultured for 24 h (E:T = 10:1) with HepG2 cells expressing the indicated PD‐L1 constructs ±siGALNT16. Right panels: CD107a^+^ CD8^+^ T cells; histograms (right) summarize mean percentages ± SD from three paired experiments. Statistical significance was assessed by paired *t*‐test (*p < 0.05, **p < 0.01, ***p < 0.001).

Subsequently, immunohistochemical staining for YY1, GALNT16, and PD‐L1 was conducted on human HCC consecutive tissue sections, and higher PD‐L1 expression levels were observed in tumor cells within tissues with high expression of YY1 and GALNT16 (Figure 5A). The statistical analysis of expression correlation revealed a positive correlation between the expression of YY1 and GALNT16 (Figure 5B), as well as between GALNT16 and PD‐L1 (Figure 5C). We observed a certain degree of overlap between the staining areas of GALNT6 and PD‐L1 in consecutive HCC sections (the same color indicates the overlapping regions) (Figure S3A). In light of the previously identified positive correlation between YY1 and PD‐L1 expression, these findings collectively suggest that the three factors are positively correlated with one another. Furthermore, mIF staining performed on paraffin‐embedded HCC tissue sections demonstrated a high degree of concordance between GALNT16 and PD‐L1 in terms of their cellular localization and expression patterns (Figure 5D). This correlation provides the spatial conditions necessary for GALNT16 to potentially regulate the glycosylation of PD‐L1 through its glycosyltransferase activity, thereby affecting the protein stability of PD‐L1. To investigate whether GALNT16 is directly involved in the glycosylation modification of PD‐L1, we coexpressed tagged PD‐L1 (Flag‐tagged) and GALNT16 (HA‐tagged) in HCC cells. Immunoprecipitation experiments revealed a direct interaction between these two proteins (Figure 5E, F). Glycosylation of PD‐L1 is crucial for its protein stability and activity, with N35, N192, N200, and N219 confirmed as the primary glycosylation sites of PD‐L1 [24, 25]. Although GALNT16 is known as a glycosyltransferase, its role in regulating these sites remains unknown. By mutating the asparagine (N) at these sites to glutamate (Q) to prevent glycosylation, we observed a significant reduction in the binding of the N219Q mutant to GALNT16 (Figure 5G), suggesting that N219 may be a potential glycosylation site for GALNT16. Furthermore, treatment with the proteasome inhibitor MG132 inhibited the inducing effect of GALNT16 on PD‐L1, but in cells overexpressing GALNT16, the ubiquitination level of PD‐L1 decreased (Figure 5H), while knockdown of GALNT16 increased the ubiquitination of PD‐L1 (Figure 5I). These results suggest that GALNT16 may promote the glycosylation of PD‐L1, thereby inhibiting its ubiquitination and subsequent protein degradation. Furthermore, we observed that in GALNT16‐knockdown HepG2 cells, the N35Q, N192Q, and N200Q PD‐L1 mutants exhibited markedly reduced glycosylation, with the hyper‐glycosylated species being most severely affected (Figure 5J). By contrast, the N219Q mutant alone abolished the hyper‐glycosylated band, indicating that N219 is indispensable for PD‐L1 hyper‐glycosylation. Once N219 was mutated, additional knockdown of GALNT16 no longer altered overall PD‐L1 glycosylation, demonstrating that GALNT16 regulates global PD‐L1 glycosylation primarily through the N219 site. This conclusion is supported by coimmunoprecipitation experiments in which GALNT16 predominantly associated with hyper‐glycosylated PD‐L1 (Figure 5E, right panel). Accordingly, in HepG2 cells with PD‐L1 knockdown, we ectopically expressed either wild‐type PD‐L1 or the N219Q mutant PD‐L1 plasmid, followed by coculture with CD8⁺ T cells to assess CD107a and IFNγ expression. Consistent with our WB results, the N219Q mutant PD‐L1 exhibited a weaker inhibitory effect on CD8⁺ T‐cell activation compared with wild‐type PD‐L1. Moreover, siGALNT16 markedly enhanced the cytotoxic activity of CD8⁺ T cells in the co‐culture with wild‐type PD‐L1‐expressing cells; however, this effect was abolished when cocultured with cells expressing the N219Q mutant PD‐L1 (Figures 5K and S3B).

### 2.6 GALNT16 is Transcriptionally Regulated by YY1

**FIGURE 6:**
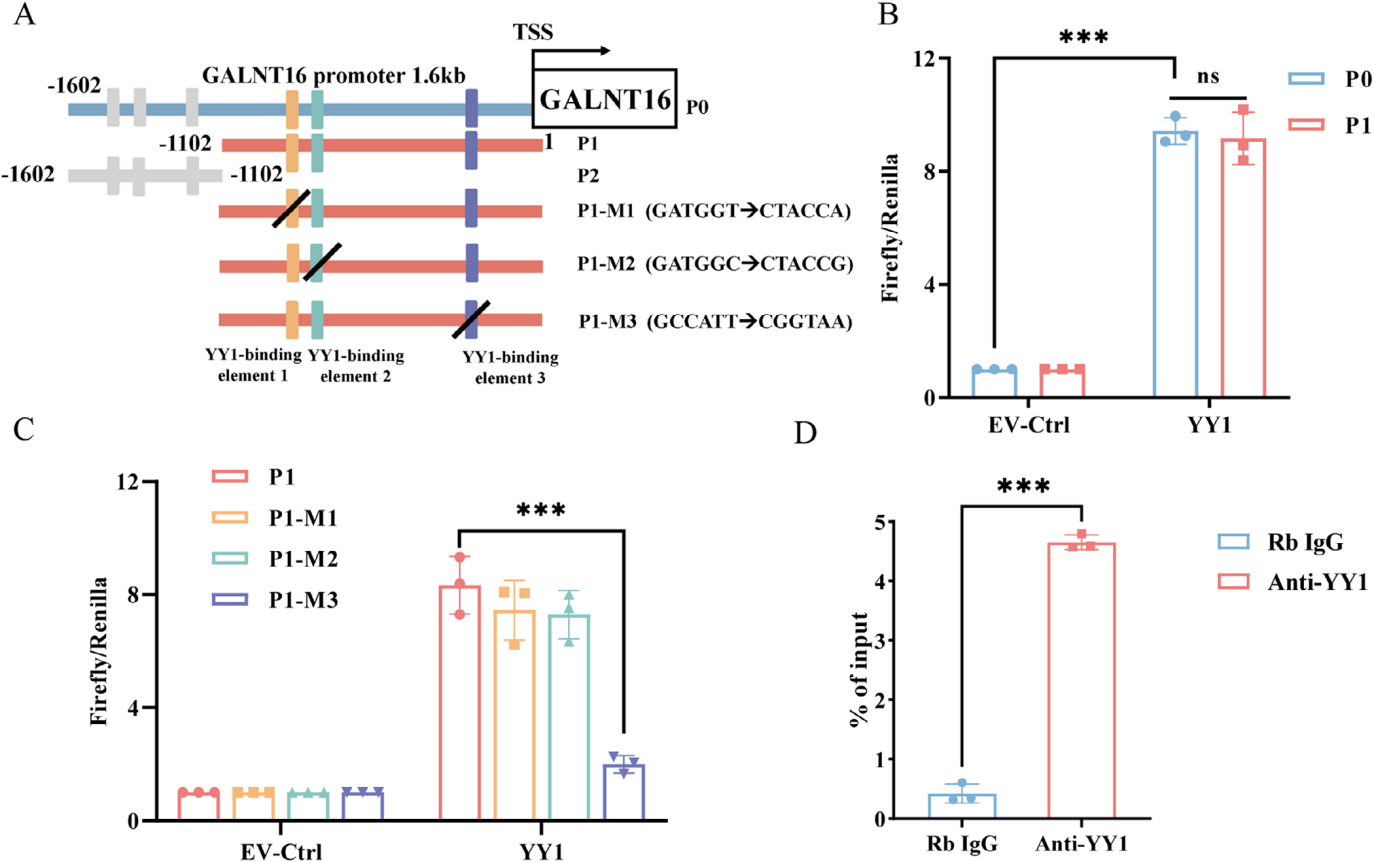
GALNT16 is transcriptionally regulated by YY1. (A) Schematic of the 1.6‐kb GALNT16 promoter (P0, −1.6 kb to +1 relative to the transcription start site; TSS) and its truncations. Six YY1 response elements (YREs) predicted by JASPAR are indicated: three in the P2 region (−1.6 to −1.1 kb) and three in the P1 region (−1.1 kb to TSS). Transition mutations (purine‐to‐purine or pyrimidine‐to‐pyrimidine) were introduced into the core sequences of the three P1 YREs, numbered from distal to proximal to the TSS, yielding constructs P1‐M1, P1‐M2, and P1‐M3. (B) Dual‐luciferase reporter assays showing luciferase activity driven by the P0 and P1 promoters in YY1‐overexpressing or control HepG2 cells. Values are mean ± SD of three independent experiments analyzed by paired t‐test; ns, not significant; ***p < 0.001. (C) Dual‐luciferase reporter assays evaluating the activity of P1 and its YRE‐mutant derivatives (P1‐M1, P1‐M2, and P1‐M3) in YY1‐overexpressing or control HepG2 cells. Mean ± SD, n = 3; paired t‐test; ***p < 0.001. (D) ChIP–qPCR quantification of YY1 occupancy at the YRE‐3 site within the P1 promoter, presented as % of input; mean ± SD, n = 3; paired t‐test; ***p < 0.001.

To ascertain whether *GALNT16* acts as a direct transcriptional target of YY1, we employed the JASPAR database to examine the 1.6 kb promoter sequence upstream of the *GALNT16* transcription start site (TSS), identifying six potential YY1 response elements (YREs) (Figure 6A). This promoter region was designated as P0. Luciferase reporter assays revealed that YY1 significantly amplified the transcriptional activity of P0. However, when the promoter segment was narrowed to the interval from −1.1 kb to the TSS, labeled as P1 (with the segment of P0 extending beyond P1 defined as P2), the transcriptional activity was found to be unaffected (Figure 6A, B). This finding implies that the three YREs located on P1 might be susceptible to YY1 regulation, whereas the three YREs on P2 potentially have no transcriptional activity regarding YY1. Further mutational analysis demonstrated that the transcriptional promotion effect of YY1 on P1 was significantly reduced, almost to baseline levels, when the most proximal YRE to the TSS (YRE‐3) was mutated (Figure 6A, C). Chromatin immunoprecipitation (ChIP)‐PCR assays provided additional evidence that YY1 can specifically bind within the YRE‐3 region (Figure 6D). Together, these findings establish *GALNT16* as a direct target gene under YY1 regulation, with YRE‐3 emerging as a pivotal element in this regulatory mechanism.

### 2.7 Targeting YY1 Enhances the Antitumor Efficacy of PD‐1 Inhibitors

Previous studies have reported that PD‐L1 proteins with more N‐glycosylation modifications exhibit increased stability, are more prone to immune escape, and have a stronger ability to bind PD‐1 [26, 27]. This affects the targeting capability of ICIs, such as anti‐PD‐1, rendering them less effective in inhibiting immune checkpoints. Therefore, we further explored whether YY1 knockdown in HCC cells makes them more susceptible to immune cell‐mediated killing and enhances the efficacy of ICIs.

**FIGURE 7:**
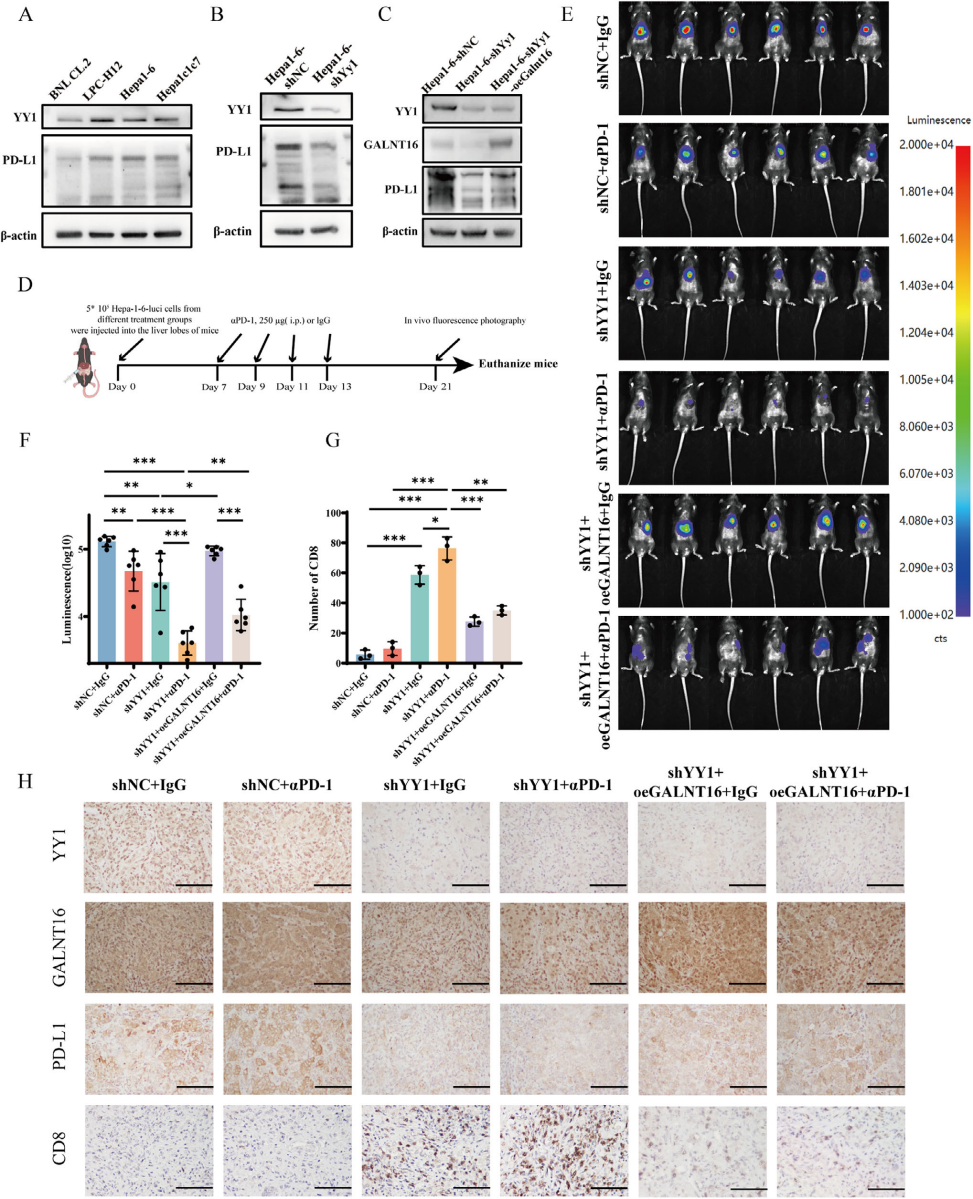
Targeting YY1 enhances the antitumor efficacy of PD‐1 blockade. (A) Basal protein expression of YY1 and PD‐L1 in mouse nontumorigenic hepatocyte line BNL CL.2 and HCC lines LPC‐H12, Hepa1‐6, and Hepa1c1c7. β‐Actin served as loading control. Representative blots from three independent experiments. (B) YY1 knockdown efficiency and concomitant PD‐L1 modulation in Hepa1‐6 cells. β‐Actin, loading control. Representative of n = 3. (C) Western blot of YY1, GALNT16, and PD‐L1 in Hepa1‐6 cells after YY1 silencing alone or combined with GALNT16 overexpression (luciferase‐tagged). β‐Actin, loading control. Representative of n = 3. (D) Timeline schematic of orthotopic Hepa1‐6‐luciferase tumor engraftment and anti‐PD‐1 or isotype IgG treatment regimen. (E) Representative in vivo bioluminescence images of mice on day 21 under the indicated treatments. Each group, n = 6. (F) Quantification of photon flux (day 21) from (E). Data are mean ± SD; unpaired t‐test; *p < 0.05, **p < 0.01, ***p < 0.001. (G) Quantification of CD8⁺ cell density in tumor sections. Each treatment group comprised six mice (n = 6); data are presented as mean ± SEM and analyzed by unpaired t‐test; *p < 0.05, **p < 0.01, ***p < 0.001. (H) Representative immunohistochemical images of YY1, GALNT16, PD‐L1, and CD8 in tumors from each group. Scale bar, 100 µm.

First, we found that the baseline expression of YY1 in mouse HCC cell lines (LPC‐H12, Hepa1‐6, and Hepa1c1c7) was higher compared with normal mouse liver cell line (BNL CL.2), with PD‐L1 expression also being higher (Figure 7A). Using the Hepa1‐6 mouse HCC cell line, we knocked down YY1 expression and observed a significant reduction in the expression of higher molecular weight glycosylated PD‐L1(Figure 7B). Then, we overexpressed GALNT16 in YY1‐knockdown cells to prepare for subsequent in vivo experiments, and we also observed that the protein level of PD‐L1 increased with GALNT16 overexpression (Figure 7C).

In the established orthotopic implantation model of HCC, mice received intraperitoneal injections of anti‐PD‐1 antibody or isotype IgG (250 µg/mouse) every 2 days for four total doses (see experimental timeline in Figure 7D). Each treatment group consisted of six mice. Bioluminescence imaging was performed at day 21 to monitor tumor progression. Tumor burden in mice was monitored using live animal imaging, and tumor size was compared across different groups based on fluorescence intensity. The results showed that the group with YY1 knockdown combined with anti‐PD‐1 treatment exhibited significant tumor growth inhibition, whereas the GALNT16 overexpression group showed accelerated tumor growth (Figures 7E, F and S4). IHC revealed that the YY1 knockdown combined with anti‐PD‐1 treatment group had significantly increased CD8^+^ T cell infiltration within the tumor, while the GALNT16 overexpression group had reduced CD8^+^ T cell infiltration (Figure 7G, H).

In summary, YY1 is highly expressed in HCC and transcriptionally regulates the expression of GALNT16. GALNT16 increases PD‐L1 stability by enhancing N‐glycosylation at the N219 site, promoting immune escape in tumors. Knocking down YY1 and combining it with anti‐PD‐1 therapy results in significant tumor shrinkage and increased CD8^+^ T cell infiltration.

## 3 Discussion

A burgeoning literature has shown that PD‐L1 molecules with heightened glycosylation exhibit significantly increased binding affinity to PD‐1, thereby bestowing increased resistance to ICIs targeting PD‐L1 [6, 28]. Accurately pinpointing PD‐L1 glycosylation levels in tumor cells is thus crucial for the effective identification of patient cohorts likely to respond to ICIs. However, this process is impeded by our incomplete grasp of the mechanisms governing PD‐L1 glycosylation. In this study, we identified the transcription factor YY1, linked to resistance to PD‐1 therapy in HCC, and demonstrated its role in fostering drug resistance by upregulating PD‐L1 expression in liver cancer cells. Specifically, YY1, functioning as a transcription factor, directly amplifies the expression of GALNT16, a glycosyltransferase, thereby enhancing PD‐L1 glycosylation in liver cancer cells and curbing PD‐L1 degradation via the ubiquitination pathway.

Currently, a variety of glycosyltransferases capable of modulating PD‐L1 glycosylation have been uncovered across diverse studies. For instance, in HCC, IL‐6/JAK1‐driven phosphorylation at Y112 of PD‐L1 engages the N‐glycosyltransferase STT3A to catalyze PD‐L1 glycosylation and sustain PD‐L1 stability [29]. In lung adenocarcinoma, β‐1,4‐galactosyltransferase 1 (B4GALT1) has been recognized as a direct catalyst of N‐linked glycosylation of PD‐L1, reinforcing its stability. Moreover, B4GALT1 has been shown to stabilize TAZ, a transcriptional coactivator of *CD274* (encoding PD‐L1), through glycosylation, thereby activating the transcription of *CD274* [30]. Additionally, β‐1,3‐N‐acetylglucosaminyl transferase (B3GNT3) has been shown to enhance PD‐L1 glycosylation, amplifying its stability and immunosuppressive function [31]. It is clear that PD‐L1 glycosylation is a multifaceted biological process that may not adhere to a simple one‐to‐one enzyme‐substrate interaction. To date, all characterized glycosylation sites on PD‐L1 are of the N‐glycosylation type, predominantly located at N35, N192, N200, and N219 [25]. Despite indications that O‐glycosylation inhibitors might induce lysosomal degradation of PD‐L1, no definitive O‐glycosylation sites have been mapped within the PD‐L1 amino acid sequence [32]. This study has revealed that polypeptide N‐acetylgalactosaminyltransferase 16 (GALNT16), also called GALNTL1, influences the overall glycosylation levels of PD‐L1. Despite GALNT16 being classified as an N‐acetylgalactosaminyltransferase, previous studies have implicated it in protein and lipid metabolism, as well as in AMPK, prolactin, and insulin/IGF signaling pathways, and it may also play a role in lipid metabolism associated with cardiovascular diseases. However, its role in mediating glycosylation modifications is not well understood. In the current study, we demonstrate that GALNT16 modulates PD‐L1 abundance by controlling glycosylation at N219, the residue linked to the hyper‐glycosylated form of the protein. While the precise molecular circuitry remains to be elucidated, the functional connection between GALNT16 activity and both the glycosylation status and steady‐state levels of PD‐L1 is now firmly supported.

The pleiotropic transcription factor YY1, another key figure in this study, was found to exhibit markedly differential expression in HCC tissues responding to and resistant to anti‐PD‐1 therapy, as identified by proteomic profiling. YY1 is highly expressed in various tumors, including HCC, and is closely associated with tumorigenesis, progression, immune evasion, and poor prognosis [33, 34, 35]. Particularly in the regulation of tumor immunity, multiple studies have implicated YY1 in the regulation of PD‐L1 expression, though the mechanisms vary [18, 36]. For example, Zhang et al. [37] have shown that YY1 acts as a direct transcription factor for *CD274*. While Xia et al. [38] have demonstrated that YY1 can indirectly upregulate the transcriptional activator c‐Fos of PD‐L1, leading to the upregulation of PD‐L1 expression. Liu et al. [39] have observed that YY1 induces PD‐L1 production by upregulating IL‐8 expression. These direct or indirect regulatory effects of YY1 on PD‐L1 typically do not extend beyond transcriptional regulation. Moreover, Song et al. [40] discovered that PD‐L1 expression is regulated by the lncRNA CASC11/E2F1 axis and identified YY1 as a transcription factor for lncRNA CASC11, but this study did not investigate the regulatory effect of YY1 on PD‐L1. In this study, we found that YY1 did not significantly impact PD‐L1 transcription but did influence posttranslational modification, occurring via GALNT16‐mediated PD‐L1 glycosylation and ubiquitin‐mediated protein degradation mechanisms.

Our study confirms the critical role of YY1 in HCC immune therapy resistance, involving PD‐L1 glycosylation and protein stability mechanisms. However, several limitations should be acknowledged. First, we have not yet deeply explored the mechanisms by which GALNT16‐mediated PD‐L1 glycosylation regulates PD‐L1 stability, nor have we identified the E2 conjugating enzymes or E3 ubiquitin ligases involved in this process. Nonetheless, experiments using the MG132 inhibitor and the glycosylation enzyme inhibitor Tunicamycin in hepatoma cells indicate that glycosylation of PD‐L1 in hepatoma cells can enhance its protein expression, consistent with reports that PD‐L1 glycosylation promotes its stability [27, 41]. Moreover, RNA‐seq analysis suggested that YY1 did not regulate the expression of certain ubiquitination pathway‐related genes (Table S3). Therefore, it remains uncertain whether the regulatory mechanism of YY1 on PD‐L1 stability requires a specific cellular context, and whether this regulatory effect may be confined to cells expressing certain ubiquitination pathway‐related proteins. Second, beyond the mechanistic insights, our study is constrained by the currently limited sample size of HCC tissues from patients receiving immunotherapy. Future studies with expanded, multicenter cohorts are necessary to validate our findings. Finally, the therapeutic potential of targeting the YY1/GALNT16 axis warrants further investigation through corresponding clinical studies, such as evaluating the combination of YY1 or GALNT16 inhibitors with anti‐PD‐1 therapy, which would be a significant supplement to this preclinical research.

Overall, our study highlights the critical function of the YY1/GALNT16/PD‐L1 axis in HCC immunotherapeutic resistance, particularly the mechanism of GALNT16‐mediated modification of PD‐L1 glycosylation, which offers a potential explanation for the discrepancy between PD‐L1 expression levels and anti‐PD‐1 immunotherapy efficacy. The notion that PD‐L1 glycosylation can be reduced by inhibiting YY1 or its downstream GALNT16, thereby improving the efficacy of anti‐PD‐1 therapies, is introduced. This therapeutic strategy has the potential to enhance the response rate of anti‐PD‐1 therapy in HCC patients and to provide new markers for identifying the potential beneficiary population.

## 4 Conclusion

Through high‐throughput proteomic analysis of HCC tissues with varying responses to anti‐PD‐1 therapy, we have identified the transcription factor YY1, associated with resistance to anti‐PD‐1 treatment. We have elucidated the mechanism by which YY1 regulates PD‐L1 expression in HCC and contributes to resistance to ICIs. Specifically, YY1 upregulates the expression of GALNT16 at the transcriptional level, increasing the level of N‐linked glycosylation of PD‐L1 and thereby stabilizing the PD‐L1 protein. The combined elevation of PD‐L1 protein levels and glycosylation levels leads to a diminished antitumor effect of CD8^+^ T cells, ultimately resulting in immunotherapy failure. This study provides a theoretical foundation for considering YY1 and GALNT16 as predictive markers for the efficacy of anti‐PD‐1 therapy in HCC and as potential combined therapeutic targets.

## 5 Materials and Methods

### 5.1 Clinical Sample Collection and Proteomic Analysis

Eight HCC tissue samples, along with their corresponding clinical data, were sourced from Sun Yat‐sen Memorial Hospital at Sun Yat‐sen University. The tissues were resected on the day of surgery, fixed in formalin, and subsequently embedded in paraffin. Pathologists verified the samples as confirmed cases of HCC. All imaging‐based assessments in the anti‐PD‐1 cohort were performed according to the iRECIST. The samples were sent to Majorbio (Shanghai, China) for high‐throughput proteomic analysis. Differentially expressed genes were identified using the R package “Limma.” The heatmap‐bubble plot was generated using the OmicStudio tools available at https://www.omicstudio.cn/tool.

### 5.2 Data Collection From Public Databases

Prognostic analysis of melanoma and glioblastoma treated with PD‐1 therapy was conducted using the Kaplan–Meier Plotter. Glycosylation‐related genes were sourced from the GlycoGene Database (https://acgg.asia/ggdb2/) and the PANTHER database (https://www.pantherdb.org/). RNA sequencing data for 239 HCC patients who had not received radiotherapy were obtained from the TCGA database. These patients were divided into two groups based on YY1 expression levels. The abundance levels of 22 immune cell types in each group were assessed using CIBERSORT. The promoter sequence of the GALNT16 gene, spanning from the TSS at +1 to −1600, was selected using the EPD database (https://epd.epfl.ch/index.php). Prediction of binding sites based on transcription factors and target gene promoter sequences was conducted using two databases: the PROMO database (http://alggen.lsi.upc.es/cgi‐bin/promo_v3/promo/promoinit.cgi?dirDB=TF_8.3) and JASPAR (https://jaspar.genereg.net/). Schematic diagram of mouse experiments (Created with BioGDP.com).

### 5.3 Cell Culture

The HepG2 and PLC/PRF/5 cell lines were purchased from the Shanghai Cell Bank of the Chinese Academy of Sciences, while the MHCC‐97H cell line was obtained from Shanghai Zhongqiao Xinzhou Company. The mouse normal liver cell line (BNL CL.2) and mouse HCC cell lines (Hepa1‐6 and Hepa1c1c7) were purchased from ATCC. The mouse HCC cell line (LPC‐H12) was obtained from Shanghai Yaji Biological Company. The medium was composed of DMEM or RPMI 1640, supplemented with 10% fetal bovine serum and 1% antibiotics (100 U/mL penicillin and 100 mg/mL streptomycin).

### 5.4 RNA Extraction, RT‐qPC, siRNA Interference, and Plasmid DNA Transfection

Total RNA was extracted from cells or tissues using RNAiso Plus, following the manufacturer's instructions. Reverse transcription was conducted with HiScript III All‐in‐one RT SuperMix Perfect for qPCR (Takara, Japan). RT‐qPCR was performed using SYBR Premix Ex Taq II (Vazyme; R333‐01) on the LightCycler 480 Real‐Time PCR System (Roche Applied Science, Mannheim, Germany). β‐Actin served as the reference control, and the relative mRNA expression levels were calculated using the 2−ΔΔCt method. Gene silencing was achieved using siRNA according to the manufacturer's guidelines. The sequences of the siRNA used are as follows:

Negative control (sense: 5′‐UUCUCCGAACGUGUCACGUTT‐3′, antisense: 5′‐ACGUGACACGUUCGGAGAATT‐3′);
siYY1 (sense: 5′‐GCCUCUCCUUUGUAUAUUATT‐3′, antisense: 5′‐UAAUAUACAAAGGAGAGGCTT‐3′);
(sense: 5′‐CGACGACUACAUUGAACAATT‐3′, antisense: 5′‐UUGUUCAAUGUAGUCGUCGTT‐3′);
siGALNT16 (sense: 5′‐GGCACUUUCUACUACUUAUGGTT‐3′, antisense: 5′‐CCAUAAGUAGUAGAAAGUGCCTT‐3′) ;
(sense: 5′‐GAAUCUUCGUGAUCGACAAGUTT‐3′, antisense: 5′‐ACUUGUCGAUCACGAAGAUUCTT‐3′);
*GALNT16* cDNA was cloned into the expression vector pcDNA3.1. DNA transfection reagent (Abclonal, RM09014P) was used for plasmid transfection.

### 5.5 Western Blotting

Total protein was extracted using RIPA lysis buffer supplemented with protease and phosphatase inhibitors. Protein concentration was measured using a BCA assay (Invitrogen; 23227). An equal amount of total protein (40 µg per lane) was separated by SDS‐PAGE on a 10% gel and subsequently transferred to a PVDF membrane (Merck Millipore; ISEQ00010). The membranes were blocked with 5% bovine serum albumin at room temperature for 1 h and then incubated overnight at 4°C with diluted primary antibodies. After washing three times with TBS containing 0.1% Tween‐20, the membranes were incubated with HRP‐conjugated secondary antibodies for 1 h at room temperature. Finally, protein signals on the membrane were detected using ECL. The antibodies employed for western blotting were as follows: YY1 (Abcam; ab109237); PD‐L1 (CST; 13684); GALNT16 (Atlas Antibodies; HPA075325; biorbyt, orb35991); HSC70 (Santa Cruz; sc‐71270); FLAG (CST; 14793); HA (Santa Cruz; sc‐57592); Ub (Santa Cruz; sc‐8017); Beta‐actin (Proteintech; 66009‐1‐Ig).

### 5.6 CHX Chase Assay

Cells were treated with 50 µM CHX (Selleck, USA), and samples were collected at 0, 4, 8, 12, and 16 h. Following collection, the samples were lysed to extract proteins for subsequent WB analysis.

### 5.7 Coimmunoprecipitation Assays and ChIP Assays

Immunoprecipitation assays were conducted following the manufacturer's instructions using FLAG‐ or HA‐conjugated magnetic beads (Fitgene, FI8201, FI8202). Briefly, HepG2 and PLC/PRF/5 cells were transfected with GALNT16‐HA (or empty vector) and PD‐L1‐Flag (or empty vector) for 48 h. The cell lysates were incubated with the magnetic beads by rotating at room temperature for 2 h. The lysates were then washed twice with lysis/wash buffer and once with deionized water. Finally, the samples were boiled for 10 min for subsequent WB analysis.

For the ChIP experiment, we adhered strictly to the instructions in the Pierce Magnetic ChIP Kit manual. Protein–DNA complexes were crosslinked in vivo with formaldehyde. The kit's reagents were used to lyse cells and extract and solubilize the crosslinked complexes. These complexes were incubated with specific antibodies and separated using Pierce Protein A/G magnetic beads. After reversing the crosslinks and digesting the proteins, purified DNA fragments were prepared for RT‐qPCR. The primer sequences used were as follows: sense: 5′‐CTACACAAGGACTCGCCGAA‐3′, antisense: 5′‐GGCCTTGAGTCACCGACTG‐3′. The antibodies employed for ChIP included YY1 (CST; 46395) and IgG (CST; 2729).

### 5.8 Dual‐Luciferase Reporter Assays

After being seeded in a 24‐well plate, cells were cotransfected with the GALNT16 promoter‐reporter gene vector, the Renilla luciferase vector, and YY1 pcDNA. Following a 48‐h incubation posttransfection, cell lysates were prepared using the Dual‐Luciferase Reporter Assay System (Vazyme; DD1205‐01). Renilla luciferase activity was utilized as an internal control for normalizing luciferase activity.

### 5.9 CD8^+^ T Cell‐Mediated Tumor Cell‐Killing Assays and Lactate Dehydrogenase Release Assay

Peripheral blood was first separated using Ficoll lymphocyte separation solution to obtain peripheral blood mononuclear cells‐enriched layer. This layer was aspirated, washed with bead buffer, and incubated with CD14‐conjugated magnetic beads at 4°C for 30 min. CD14‐negative cells were then selected and further incubated with CD8‐conjugated magnetic beads to isolate CD8‐positive lymphocytes. The isolated cells were added to a 24‐well plate precoated with CD3, supplemented with CD28, and cultured in T cell culture medium.

After coculturing the isolated CD8^+^ T lymphocytes with HCC cells for 24 h, the suspended lymphocytes were collected by centrifugation. These cells were incubated with flow cytometry antibodies CD8 (Biolegend; 344730), CD107a (Biolegend; 328606), and IFNγ (eBioscience; 17‐7311‐82) at room temperature for 30 min, followed by flow cytometric analysis.

Lactate dehydrogenase (LDH) is a stable cytoplasmic enzyme released extracellularly upon membrane damage. Its activity is quantified by measuring absorbance with a microplate reader following substrate reaction, serving as an indicator of cellular injury or lysis (Promega; G1780). Target cells were plated in 96‐well plates and cocultured with effector cells (CD8⁺ T cells) at specified effector‐to‐target (E:T) ratios: 1:1, 10:1, 20:1, and 40:1. After incubation, culture supernatants were centrifuged and transferred to fresh 96‐well plates. An equal volume of LDH detection reagent was added to each well, followed by 30‐min incubation at room temperature protected from light. Absorbance was measured at 490 nm using a microplate reader.

Cytotoxicity was measured by LDH release assay and calculated as: % specific cytotoxicity = [(experimental release − effector spontaneous release − target spontaneous release)/(target maximum release − target spontaneous release)] × 100. Data shown as mean ± SD (*n* = 3).

### 5.10 IHC and Immunofluorescence Assays

Tissue sections were obtained from the Sun Yat‐sen Memorial Hospital of Sun Yat‐sen University, utilizing samples from 37 postsurgical HCC patients who received anti‐PD‐1 therapy. The HCC tissues were formalin‐fixed and paraffin‐embedded (FFPE). Similarly, HCC tissues from experimental animals were also formalin‐fixed, paraffin‐embedded, and sectioned. Following deparaffinization and rehydration, antigen retrieval was conducted in Tris–EDTA buffer (pH 9.0) at elevated temperatures. After cooling, endogenous peroxidase activity was inhibited with 3% hydrogen peroxide, and sections were blocked with 5% goat serum. Primary antibodies were incubated overnight at 4°C, followed by incubation with secondary antibodies at 37°C for 30 min the following day. DAB was employed for chromogenic detection, and hematoxylin was used for nuclear counterstaining. Three random fields per section were selected, and staining scores were calculated using ImageJ to determine the average optical density.

For mIF, sections were incubated with primary antibodies from different species overnight at 4°C, followed by incubation with fluorescent secondary antibodies (Invitrogen; A21203 and A21206) the next day, and DAPI for nuclear staining. The antibodies employed for immunohistochemical and immunofluorescence were as follows: YY1 (Abcam, ab109237; Proteintech, 66248‐1‐Ig); PD‐L1 (CST; 13684); GALNT16 (Atlas Antibodies, HPA059136; biorbyt, orb183836); CD8 (ZS, ZA‐0508‐0.2; Proteintech, 66868‐1‐Ig); and CD3 (Proteintech; 60181‐1‐Ig).

### 5.11 Animal Experiments

Six‐week‐old C57BL/6 mice were purchased from GemPharmatech company and divided into six groups, with six mice in each group. A total of 25 µL of a solution containing 500,000 Hepa1‐6 cells mixed with PBS and Matrigel at a 1:1 ratio was injected subcutaneously into the liver lobes of each mouse. One week after the injection, tumor formation was assessed. Mice then received intraperitoneal injections of 250 µg of anti‐PD‐1 monoclonal antibody (Selleck, A2122) or IgG isotype control (Selleck; A2123) every 2 days for a total of three injections. Tumor changes were monitored using small animal in vivo imaging following the three injections. We ordered lentiviruses with an HA tag for the overexpression of Galnt16 (NCBI Reference Sequence: NM_001081421.2) and lentiviruses for the knockdown of YY1 from GenePharma (Shanghai). The target sequence for YY1 knockdown is GCCTCTCCTTTGTATATTATT.

### 5.12 Statistical Analyses

Statistical analyses were performed using R (v4.0.5). Continuous variables are presented as means ± standard deviations, and comparisons between continuous variables in the control and treatment groups were conducted using either the *t*‐test or the Wilcoxon test. Categorical variables were analyzed using the chi‐square test, with Fisher's exact test applied for samples with fewer than 40 observations. Correlation analysis of continuous variables was performed using Pearson correlation coefficients. Group comparisons of count data were conducted using the chi‐square test. PFS was estimated using the Kaplan–Meier method. A two‐tailed *p* value of <0.05 was considered statistically significant.

## Author Contributions

Shu‐sheng Lin carried out majority of the experiments and wrote the draft of the paper. Gang Xiao provided guidance on the experiment and revised the draft. Qin‐qin Liu participated in writing the paper and assisted with some of the experiments. Jia‐hao Xue assisted in completing the animal‐related experiments. Zhi‐jun Chen collected data related to clinical information. Hong‐hua Zhang guided the completion of bioinformatics analyses. Xiang‐ping Zhu, Keng‐long Huang, Cai‐ni Yang, and Ke Zhu assisted with some of the experiments. Hao‐ming Lin and Rui Zhang provided clinical samples, devised the experimental plan, and contributed to writing the paper. All authors have read and approved the final manuscript.

## Funding

This work was supported by the Special Research Foundation of the National Nature Science Foundation of China (82273476), the Guangdong Basic and Applied Basic Research Foundation (2023A1515010188); Fundamental Research Funds for the Central Universities, Sun Yat‐sen University (24xkjc035), Guangzhou Key Laboratory of Precise Diagnosis and Treatment of Biliary Tract Cancer (202201020375), Grant [2013]163 from Key Laboratory of Malignant Tumor Molecular Mechanism and Translational Medicine of Guangzhou Bureau of Science and Information Technology, Grant KLB09001 from the Key Laboratory of Malignant Tumor Gene Regulation and Target Therapy of Guangdong Higher Education Institutes, and Grant from Guangdong Science and Technology Department (2015B050501004, 2017B030314026). Fundamental Research Funds for the Central Universities, Clinical Research 5010 Program, Sun Yat‐sen University (Grant number 2018008).

## Ethics Statement

This manuscript has obtained informed consent from the patients. The studies involving human participants were obtained and approved by Ethics committees of Sun Yat‐Sen Memorial Hospital, Sun Yat‐Sen University (Protocol Code: SYSKY‐2023‐471‐01). This animal experiment design and methods were approved by the Guangzhou Yongnuo Biological Animal Welfare Ethics Committee, approval number: IACUC‐AEWC‐F240423023.

## Conflicts of Interest

The authors declare no conflicts of interest.

## Supporting information




**Figure S1**: Association between MPLKIP expression and survival in anti‐PD‐1‐treated patients across multiple cancer types. (A–C) Kaplan–Meier curves for overall survival (OS) or progression‐free survival (PFS) based on MPLKIP expression levels. (A) OS in glioblastoma patients (KM‐Plotter database cohort, *n* = 28). (B) OS in melanoma patients (KM‐Plotter database cohort, *n* = 334). (C) PFS in hepatocellular carcinoma (HCC) patients (our institutional cohort, *n* = 37). *p* Values were calculated by the log‐rank test.
**Figure S2**: Validation of GALNT16‐mediated regulation of PD‐L1 expression. (A) RT‐qPCR analysis of *GALNT16* and *PD‐L1* mRNA levels in the indicated cell lines with GALNT16 knockdown (siGALNT16) or overexpression (oeGALNT16). Data were normalized to *ACTB* (2^–ΔΔCt^) and are presented as mean ± SD (*n* = 3); Significance was determined by unpaired *t*‐test (ns, not significant; **p < 0.01, ***p < 0.001). (B) Western blot analysis validating the efficiency of YY1 and GALNT16 manipulation and its effect on PD‐L1 protein expression. HSC70 was used as a loading control. Blots are representative of three independent experiments.
**Figure S3**: Spatial concordance between GALNT16 and PD‐L1 expression and its functional consequence on CD8^+^ T cells. (A) Representative immunohistochemical images of serial tissue sections stained for GALNT16 and PD‐L1, respectively. Five distinct colored circles (green, yellow, blue, brown, red) highlight corresponding regions across the two sections, demonstrating a consistent positive trend between GALNT16 and PD‐L1 expression levels at different anatomical sites. (B) Flow cytometric analysis of CD8⁺ T cell function after a 24‐h coculture with HepG2 cells expressing the indicated PD‐L1 constructs, with or without GALNT16 knockdown (E:T ratio = 10:1). The left panel shows representative flow cytometry plots, and the right panel summarizes the percentages of IFN‐γ^+^ CD8^+^ T cells from three independent experiments (mean ± SD). Statistical significance was determined by a paired *t*‐test (*p < 0.05, **p < 0.01, ***p < 0.001).
**Figure S4**: GALNT16 overexpression attenuates the enhanced anti‐PD‐1 response induced by YY1 knockdown. Macroscopic images of dissected livers from all mice (*n* = 6 per group) bearing Hepa1‐6 tumors, showcasing the combined effects of genetic perturbation (shNC, shYY1, or shYY1 + oeGALNT16) and immunotherapy (anti‐PD‐1 or isotype control IgG). The tan‐to‐brown colored areas represent tumor foci.
**Table S1**: The clinicopathological details of HCC patients who received adjuvant anti‐PD‐1 treatment with high or low YY1 and PD‐L1 expression in our cohort.
**Table S2**: The clinicopathological details of HCC patients who received adjuvant conventional (nonimmunotherapy) treatment with high or low YY1 expression in our cohort.
**Table S3**: Differentially expressed genes obtained by RNA‐seq analysis were subjected to KEGG analysis and related enrichment pathways were obtained.

## Data Availability

Some or all data, models, or code generated or used during the study are available from the corresponding author by request.
