# Supplementary material for: Yin Yang 1 Promotes Antiprogrammed Cell Death‐1 Resistance in Hepatocellular Carcinoma through Polypeptide N‐Acetylgalactosaminyltransferase 16‐Mediated Glycosylation of Programmed Death Ligand‐1
Source: MedComm (2020). 2025 Nov 26;6(12):e70504. doi: 10.1002/mco2.70504 (PMC12658286; doi:10.1002/mco2.70504)
Supplement: Supplementary file 1 — Figure S1: Association between MPLKIP expression and survival in anti‐PD‐1‐treated patients across multiple cancer types. (A–C) Kaplan–Meier curves for overall survival (OS) or progression‐free survival (PFS) based on MPLKIP expression levels. (A) OS in glioblastoma patients (KM‐Plotter database cohort, n = 28). (B) OS in melanoma patients (KM‐Plotter database cohort, n = 334). (C) PFS in hepatocellular carcinoma (HCC) patients (our institutional cohort, n = 37). p Values were calculated by the log‐rank test. Figure S2: Validation of GALNT16‐mediated regulation of PD‐L1 expression. (A) RT‐qPCR analysis of GALNT16 and PD‐L1 mRNA levels in the indicated cell lines with GALNT16 knockdown (siGALNT16) or overexpression (oeGALNT16). Data were normalized to ACTB (2–ΔΔCt) and are presented as mean ± SD (n = 3); Significance was determined by unpaired t‐test (ns, not significant; **p < 0.01, ***p < 0.001). (B) Western blot analysis validating the efficiency of YY1 and GALNT16 manipulation and its effect on PD‐L1 protein expression. HSC70 was used as a loading control. Blots are representative of three independent experiments. Figure S3: Spatial concordance between GALNT16 and PD‐L1 expression and its functional consequence on CD8+ T cells. (A) Representative immunohistochemical images of serial tissue sections stained for GALNT16 and PD‐L1, respectively. Five distinct colored circles (green, yellow, blue, brown, red) highlight corresponding regions across the two sections, demonstrating a consistent positive trend between GALNT16 and PD‐L1 expression levels at different anatomical sites. (B) Flow cytometric analysis of CD8⁺ T cell function after a 24‐h coculture with HepG2 cells expressing the indicated PD‐L1 constructs, with or without GALNT16 knockdown (E:T ratio = 10:1). The left panel shows representative flow cytometry plots, and the right panel summarizes the percentages of IFN‐γ+ CD8+ T cells from three independent experiments (mean ± SD). Statistical significance was [file MCO2-6-e70504-s001.docx]

**Yin Yang 1 Promotes anti-programmed Cell Death-1 Resistance in Hepatocellular Carcinoma Through Polypeptide N-Acetylgalactosaminyltransferase 16-Mediated Glycosylation of Programmed Death Ligand-1**

**Author:** Shu-sheng Lin^#1,2^, Gang Xiao^#3,4^, Qin-qin Liu^#5^, Jia-hao Xue^1,2^, Zhi-jun Chen^1,2^, Hong-hua Zhang^1,2^, Xiang-ping Zhu^6^, Keng-long Huang^1,2^, Cai-ni Yang^1,2^, Ke Zhu^1,2^, Hao-ming Lin^*1,2^, Rui Zhang^*1,2^

^1^Department of Biliary-Pancreatic Surgery, Sun Yat-sen Memorial Hospital, Sun Yat-sen University, Guangzhou, China.

^2^Guangzhou Key Laboratory of Precise Diagnosis and Treatment of Biliary Tract Cancer, Sun Yat-sen Memorial Hospital, Sun Yat-sen University, Guangzhou, China.

^3^Department of Thoracic Surgery, Guangzhou First People's Hospital, Guangzhou medical University

^4^Center for Medical Research on Innovation and Translation, Guangzhou First People's Hospital, Guangzhou medical University

^5^Department of Hepatobiliary Surgery, Southwest Hospital, Third Military Medical University (Army Medical University), Chongqing, China

^6^Department of Oncology, Sun Yat-Sen Memorial Hospital, Sun Yat-Sen University, 510120, Guangzhou, China.

**# Contributed equally**

***Correspondence:**

Hao-ming Lin [(linhaom@mail.sysu.edu.cn)](mailto:(linhaom@mail.sysu.edu.cn)) | Rui Zhang (zhangr95@mail.sysu.edu.cn)

**Supplementary figures & legends**

**
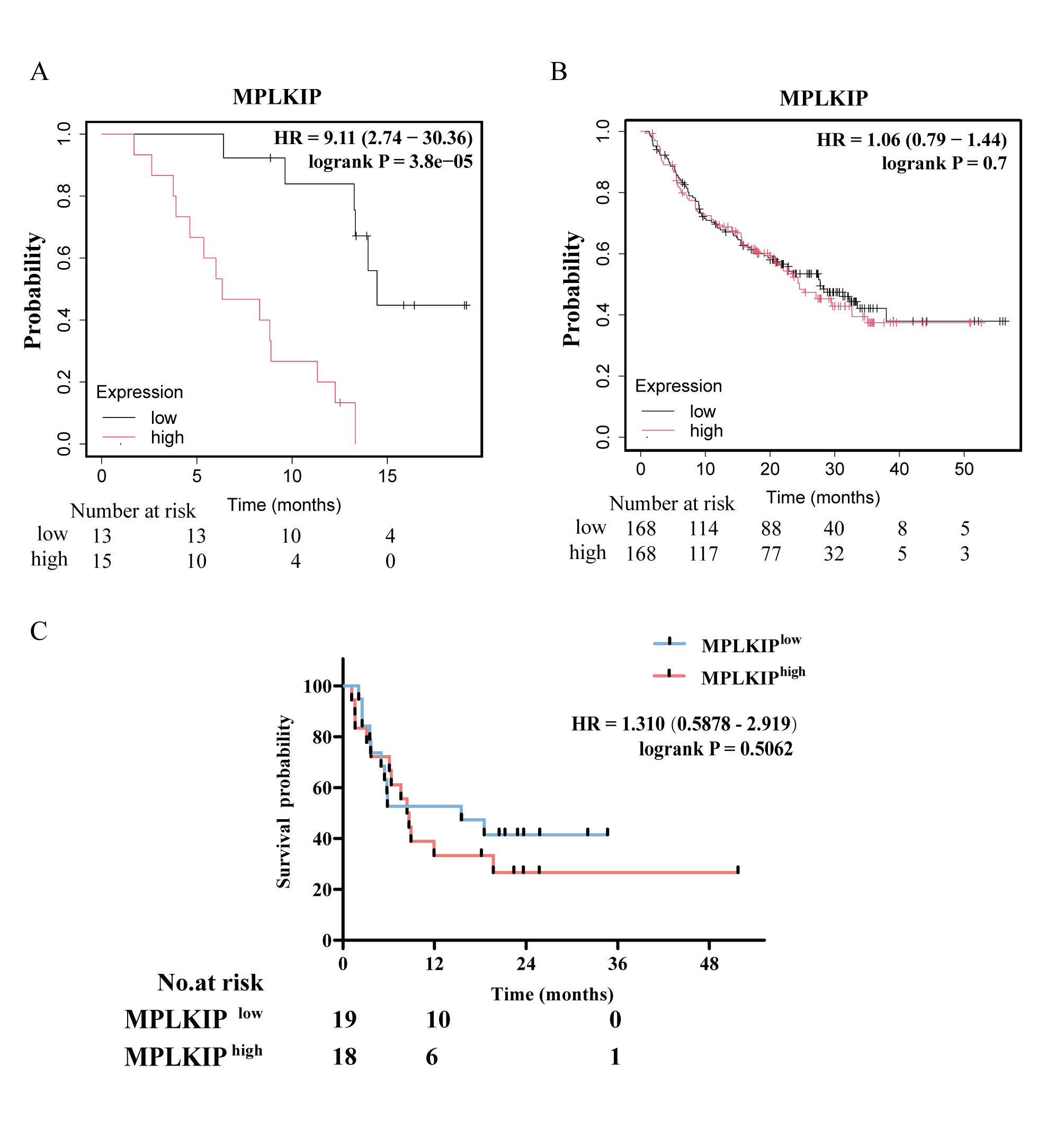
**

Figure S1. Association between MPLKIP expression and survival in anti-PD-1-treated patients across multiple cancer types.

A-C. Kaplan-Meier curves for overall survival (OS) or progression-free survival (PFS) based on MPLKIP expression levels. A. OS in glioblastoma patients (KM-Plotter database cohort, n=28). B. OS in melanoma patients (KM-Plotter database cohort, n=334). C. PFS in hepatocellular carcinoma (HCC) patients (our institutional cohort, n=37). *P* values were calculated by the log-rank test.


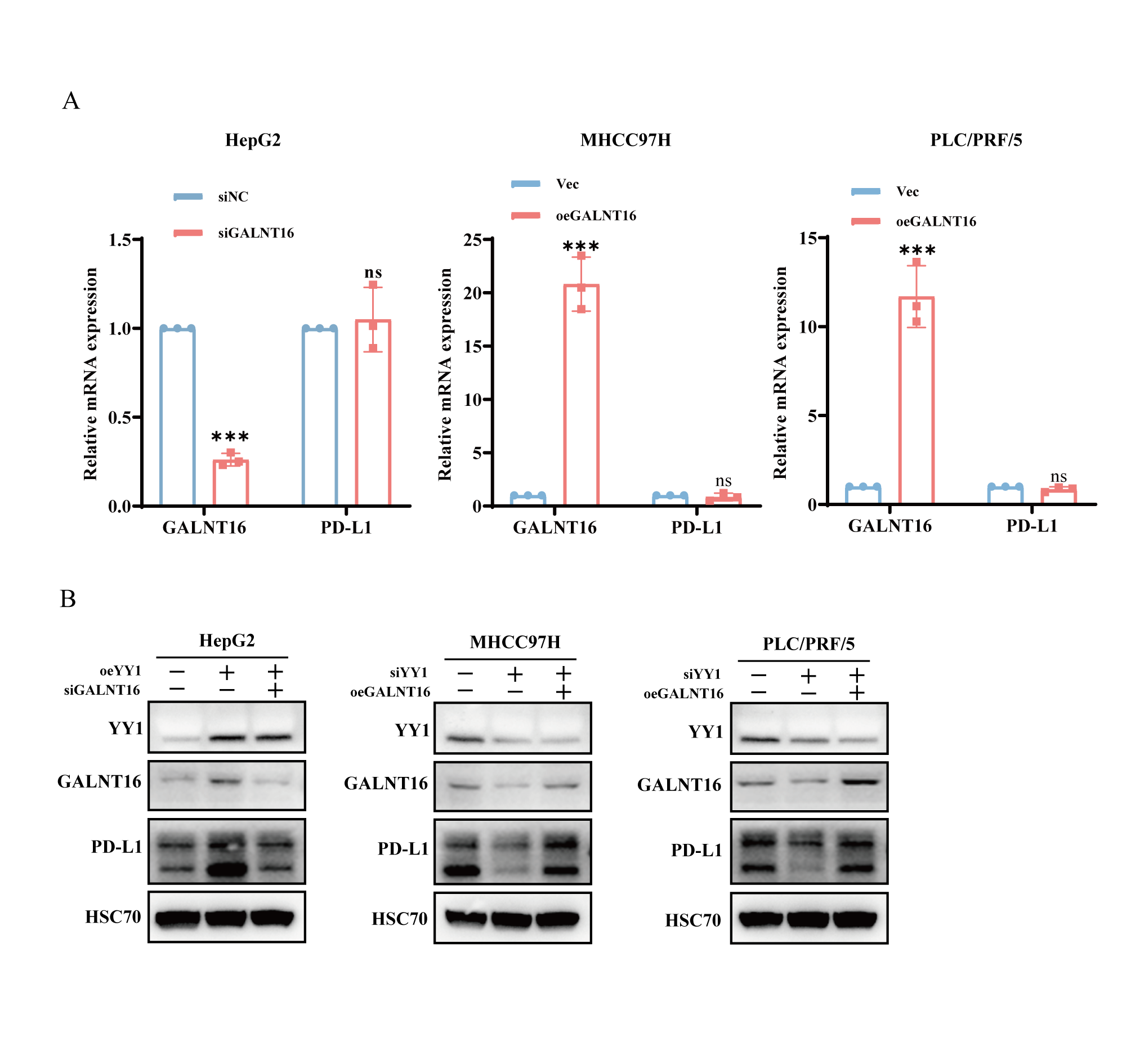


Figure S2. Validation of GALNT16-mediated regulation of PD-L1 expression.

A. RT-qPCR analysis of *GALNT16* and *PD-L1* mRNA levels in the indicated cell lines with GALNT16 knockdown (siGALNT16) or overexpression (oeGALNT16). Data were normalized to *ACTB* (2^–ΔΔCt^) and are presented as mean ± SD (n=3); Significance was determined by unpaired t-test (ns, not significant; ***P* < 0.01, ****P* < 0.001).

B. Western blot analysis validating the efficiency of YY1 and GALNT16 manipulation and its effect on PD-L1 protein expression. HSC70 was used as a loading control. Blots are representative of three independent experiments.


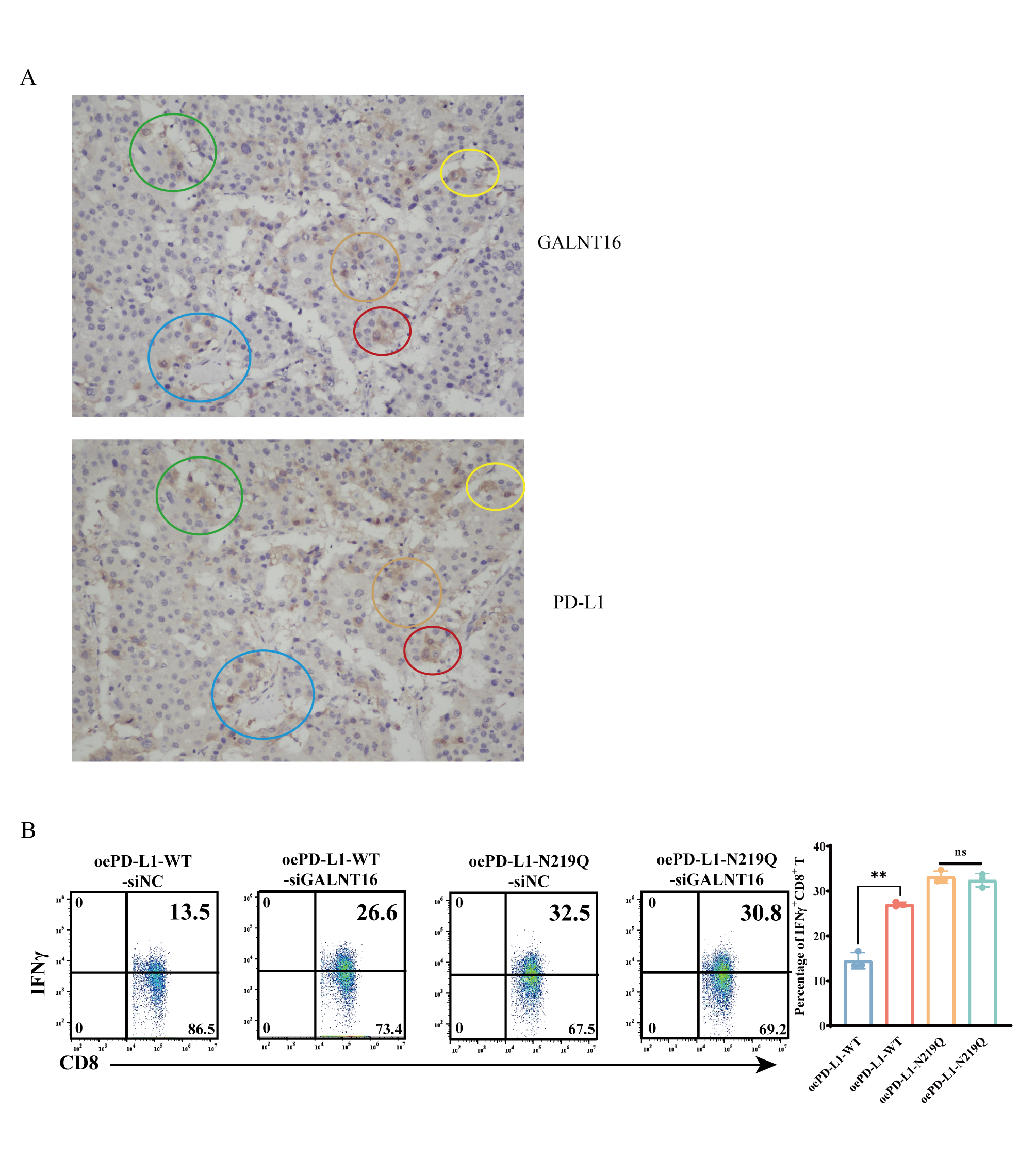


Figure S3. Spatial concordance between GALNT16 and PD-L1 expression and its functional consequence on CD8^+^ T cells.

A. Representative immunohistochemical images of serial tissue sections stained for GALNT16 and PD-L1, respectively. Five distinct colored circles (green, yellow, blue, brown, red) highlight corresponding regions across the two sections, demonstrating a consistent positive trend between GALNT16 and PD-L1 expression levels at different anatomical sites.

B. Flow-cytometric analysis of CD8⁺ T cell function after a 24-hour co-culture with HepG2 cells expressing the indicated PD-L1 constructs, with or without GALNT16 knockdown (E:T ratio = 10:1).. The left panel shows representative flow cytometry plots, and the right panel summarizes the percentages of IFN-γ^+^ CD8^+^ T cells from three independent experiments (mean ± SD). Statistical significance was determined by a paired t-test (**P* < 0.05, ***P* < 0.01, ****P* < 0.001).


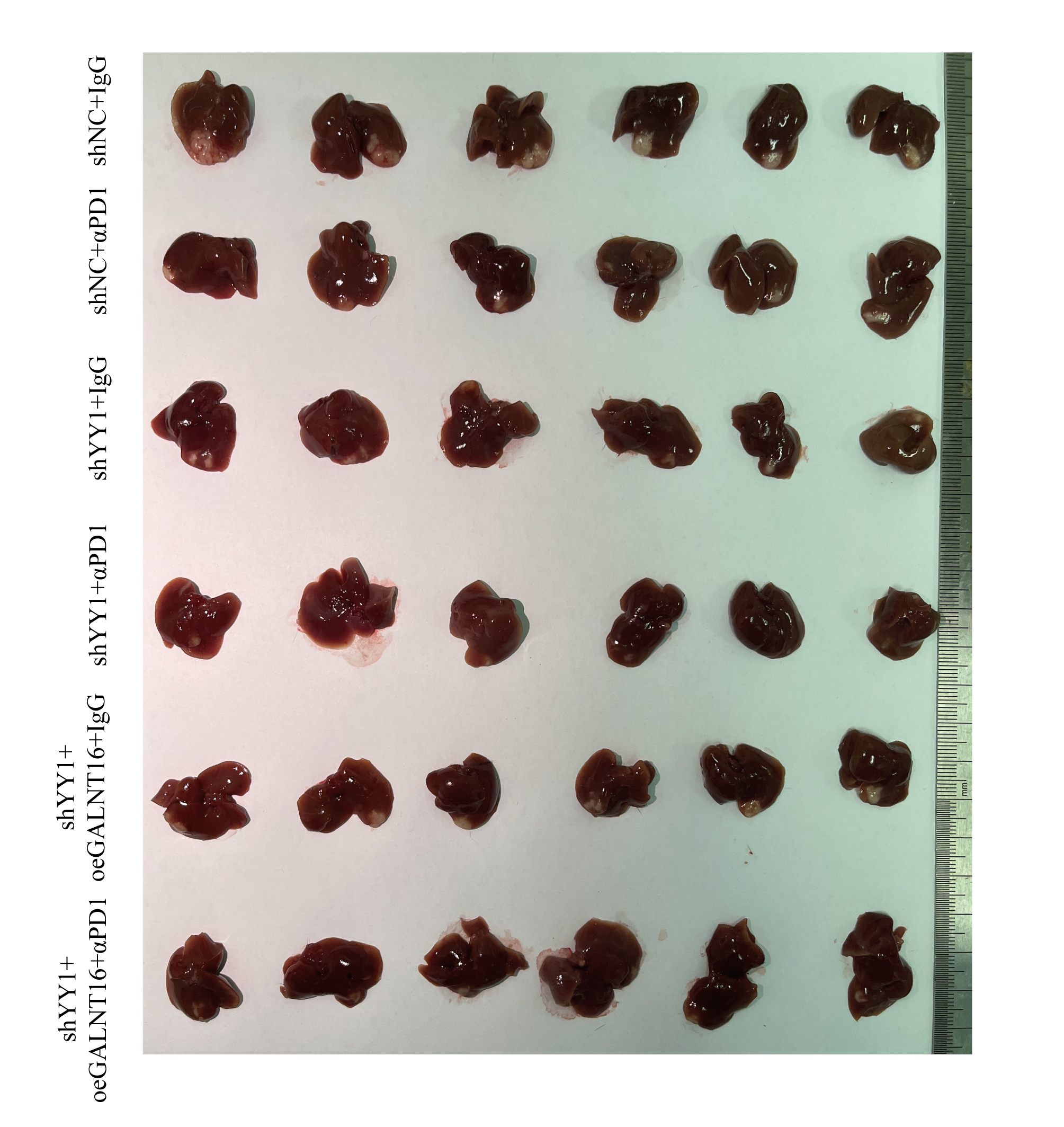


Figure S4. GALNT16 overexpression attenuates the enhanced anti-PD-1 response induced by YY1 knockdown.

Macroscopic images of dissected livers from all mice (n=6 per group) bearing Hepa1-6 tumors, showcasing the combined effects of genetic perturbation (shNC, shYY1, or shYY1 + oeGALNT16) and immunotherapy (anti-PD-1 or isotype control IgG). The tan-to-brown colored areas represent tumor foci.

Table S1. The clinicopathological details of HCC patients who received adjuvant anti-PD-1 treatment with high or low YY1 and PD-L1 expression in our cohort.

| Variables | All cases | YY1 | |  | PD-L1 | |  |
| --- | --- | --- | --- | --- | --- | --- | --- |
|  |  | Low | High | *P-*value | Low | High | *P-*value |
| Gender |  |  |  |  |  |  |  |
| Male | 34 | 22 | 12 |  | 16 | 18 |  |
| Female | 3 | 2 | 1 | >0.9999 | 0 | 3 | 0.2432 |
| Age(years) |  |  |  |  |  |  |  |
| ＜60 | 31 | 19 | 12 |  | 14 | 17 |  |
| ≥60 | 6 | 5 | 1 | 0.3945 | 2 | 4 | 0.6796 |
| HBV |  |  |  |  |  |  |  |
| Negative | 5 | 4 | 1 |  | 2 | 3 |  |
| Positive | 32 | 20 | 12 | 0.6378 | 14 | 18 | >0.9999 |
| Cirrhosis |  |  |  |  |  |  |  |
| No | 10 | 8 | 2 |  | 5 | 5 |  |
| Yes | 27 | 16 | 11 | 0.4395 | 11 | 16 | 0.7165 |
| AFP(ng/ml) |  |  |  |  |  |  |  |
| ＜400 | 22 | 14 | 8 |  | 12 | 10 |  |
| ≥400 | 15 | 10 | 5 | >0.9999 | 4 | 11 | 0.1757 |
| TNM stage |  |  |  |  |  |  |  |
| Ⅰ-Ⅱ | 15 | 11 | 4 |  | 7 | 8 |  |
| Ⅲ-Ⅳ | 22 | 13 | 9 | 0.4908 | 9 | 13 | 0.7486 |
| Therapy |  |  |  |  |  |  |  |
| IT | 12 | 9 | 3 |  | 7 | 5 |  |
| IT+CT | 13 | 8 | 5 |  | 5 | 8 |  |
| IT+CT+TT | 12 | 7 | 5 | 0.754 | 4 | 8 | 0.496 |

Table S2. The clinicopathological details of HCC patients who received adjuvant conventional (non-immunotherapy) treatment with high or low YY1 expression in our cohort.

| Variables | All cases | YY1 | |  |
| --- | --- | --- | --- | --- |
|  |  | Low | High | *P-*value |
| Gender |  |  |  |  |
| Male | 37 | 24 | 13 |  |
| Female | 5 | 2 | 3 | 0.3520 |
| Age(years) |  |  |  |  |
| ＜60 | 29 | 19 | 10 |  |
| ≥60 | 13 | 7 | 6 | 0.5097 |
| HBV |  |  |  |  |
| Negative | 6 | 3 | 3 |  |
| Positive | 36 | 23 | 13 | 0.6580 |
| Cirrhosis |  |  |  |  |
| No | 16 | 11 | 5 |  |
| Yes | 26 | 15 | 11 | 0.5299 |
| AFP(ng/ml) |  |  |  |  |
| ＜400 | 24 | 15 | 9 |  |
| ≥400 | 18 | 11 | 7 | >0.9999 |
| TNM stage |  |  |  |  |
| Ⅰ-Ⅱ | 11 | 9 | 2 |  |
| Ⅲ-Ⅳ | 31 | 17 | 14 | 0.1579 |
| Therapy |  |  |  |  |
| Surgery | 13 | 12 | 4 |  |
| Surgery + CT | 29 | 14 | 12 | 0.2064 |

Table S3. Differentially expressed genes obtained by RNA-seq analysis were subjected to KEGG analysis and related enrichment pathways were obtained.

| ID | Description | GeneRatio | BgRatio | *P*-value | *P*-adjust | qvalue | geneID | Count |
| --- | --- | --- | --- | --- | --- | --- | --- | --- |
| hsa04978 | Mineral absorption | 9/135 | 61/8846 | 0.0000 | 0.0001 | 0.0001 | MT1G/MT2A/MT1E/MT1M/MT1F/MT1X/MT1B/ATP2B3/MT1A | 9 |
| hsa00900 | Terpenoid backbone biosynthesis | 5/135 | 23/8846 | 0.0000 | 0.0025 | 0.0025 | MVD/MVK/HMGCS1/ACAT2/IDI1 | 5 |
| hsa00100 | Steroid biosynthesis | 3/135 | 20/8846 | 0.0033 | 0.2632 | 0.2632 | LSS/EBP/MSMO1 | 3 |
| hsa00280 | Valine, leucine and isoleucine degradation | 4/135 | 48/8846 | 0.0060 | 0.3618 | 0.3618 | BCAT1/HMGCS1/ACAT2/IL4I1 | 4 |
| hsa01210 | 2-Oxocarboxylic acid metabolism | 3/135 | 33/8846 | 0.0136 | 0.6295 | 0.6295 | BCAT1/OGDHL/GPT | 3 |
| hsa04925 | Aldosterone synthesis and secretion | 5/135 | 98/8846 | 0.0168 | 0.6295 | 0.6295 | STAR/PRKD1/ATP2B3/ADCY1/CALML4 | 5 |
| hsa04371 | Apelin signaling pathway | 6/135 | 140/8846 | 0.0201 | 0.6295 | 0.6295 | NOTCH3/PPARGC1A/SPP1/GNG4/ADCY1/CALML4 | 6 |
| hsa05416 | Viral myocarditis | 4/135 | 69/8846 | 0.0209 | 0.6295 | 0.6295 | SGCA/FYN/HLA-B/LAMA2 | 4 |
| hsa01200 | Carbon metabolism | 5/135 | 116/8846 | 0.0321 | 0.7990 | 0.7990 | OGDHL/ACAT2/ACSS1/GPT/ALDOB | 5 |
| hsa00670 | One carbon pool by folate | 2/135 | 20/8846 | 0.0367 | 0.7990 | 0.7990 | ALDH1L1/ALDH1L2 | 2 |
| hsa00565 | Ether lipid metabolism | 3/135 | 50/8846 | 0.0405 | 0.7990 | 0.7990 | PLAAT3/PLA2G2C/TMEM86B | 3 |
| hsa00592 | alpha-Linolenic acid metabolism | 2/135 | 26/8846 | 0.0592 | 0.7990 | 0.7990 | PLAAT3/PLA2G2C | 2 |
| hsa00650 | Butanoate metabolism | 2/135 | 27/8846 | 0.0633 | 0.7990 | 0.7990 | HMGCS1/ACAT2 | 2 |
| hsa04820 | Cytoskeleton in muscle cells | 7/135 | 232/8846 | 0.0644 | 0.7990 | 0.7990 | SGCA/COMP/VIM/LAMA2/FBN1/FMNL1/TCAP | 7 |
| hsa04966 | Collecting duct acid secretion | 2/135 | 28/8846 | 0.0675 | 0.7990 | 0.7990 | SLC4A1/ATP6V0A4 | 2 |
| hsa05034 | Alcoholism | 6/135 | 188/8846 | 0.0677 | 0.7990 | 0.7990 | H2AC16/H4C11/GNG4/H2AC20/CALML4/H2AC4 | 6 |
| hsa04014 | Ras signaling pathway | 7/135 | 238/8846 | 0.0717 | 0.7990 | 0.7990 | PLAAT3/FLT4/PLA2G2C/RASSF5/GNG4/PDGFRA/CALML4 | 7 |
| hsa04744 | Phototransduction | 2/135 | 29/8846 | 0.0718 | 0.7990 | 0.7990 | CNGB1/CALML4 | 2 |
| hsa04613 | Neutrophil extracellular trap formation | 6/135 | 192/8846 | 0.0733 | 0.7990 | 0.7990 | CLCN4/MPO/H2AC16/H4C11/H2AC20/H2AC4 | 6 |
| hsa00591 | Linoleic acid metabolism | 2/135 | 30/8846 | 0.0762 | 0.7990 | 0.7990 | PLAAT3/PLA2G2C | 2 |
| hsa04072 | Phospholipase D signaling pathway | 5/135 | 149/8846 | 0.0775 | 0.7990 | 0.7990 | FYN/DNM1/GRM2/ADCY1/PDGFRA | 5 |
| hsa00630 | Glyoxylate and dicarboxylate metabolism | 2/135 | 31/8846 | 0.0807 | 0.7990 | 0.7990 | ACAT2/ACSS1 | 2 |
| hsa04510 | Focal adhesion | 6/135 | 203/8846 | 0.0902 | 0.7990 | 0.7990 | FYN/FLT4/COMP/LAMA2/SPP1/PDGFRA | 6 |
| hsa04145 | Phagosome | 5/135 | 157/8846 | 0.0920 | 0.7990 | 0.7990 | MPO/TUBA1A/COMP/ATP6V0A4/HLA-B | 5 |
| hsa04514 | Cell adhesion molecules | 5/135 | 157/8846 | 0.0920 | 0.7990 | 0.7990 | CLDN2/JAM3/HLA-B/SPN/NEO1 | 5 |
| hsa04725 | Cholinergic synapse | 4/135 | 115/8846 | 0.0988 | 0.7990 | 0.7990 | FYN/KCNJ12/GNG4/ADCY1 | 4 |
| hsa04670 | Leukocyte transendothelial migration | 4/135 | 116/8846 | 0.1012 | 0.7990 | 0.7990 | MMP2/CLDN2/JAM3/RASSF5 | 4 |
| hsa04724 | Glutamatergic synapse | 4/135 | 116/8846 | 0.1012 | 0.7990 | 0.7990 | HOMER2/GRM2/GNG4/ADCY1 | 4 |
| hsa03082 | ATP-dependent chromatin remodeling | 4/135 | 117/8846 | 0.1036 | 0.7990 | 0.7990 | H2AC16/YY1/H2AC20/H2AC4 | 4 |
| hsa04015 | Rap1 signaling pathway | 6/135 | 212/8846 | 0.1054 | 0.7990 | 0.7990 | FLT4/RASSF5/PRKD1/ADCY1/PDGFRA/CALML4 | 6 |
| hsa01230 | Biosynthesis of amino acids | 3/135 | 75/8846 | 0.1066 | 0.7990 | 0.7990 | BCAT1/GPT/ALDOB | 3 |
| hsa00250 | Alanine, aspartate and glutamate metabolism | 2/135 | 37/8846 | 0.1090 | 0.7990 | 0.7990 | GPT/IL4I1 | 2 |
| hsa04022 | cGMP-PKG signaling pathway | 5/135 | 166/8846 | 0.1099 | 0.7990 | 0.7990 | CNGB1/OPRD1/ATP2B3/ADCY1/CALML4 | 5 |
| hsa04919 | Thyroid hormone signaling pathway | 4/135 | 122/8846 | 0.1160 | 0.7990 | 0.7990 | SLC16A2/SLC16A10/MED12L/NOTCH3 | 4 |
| hsa04935 | Growth hormone synthesis, secretion and action | 4/135 | 122/8846 | 0.1160 | 0.7990 | 0.7990 | MRAP2/SSTR1/IGFBP3/ADCY1 | 4 |
| hsa05163 | Human cytomegalovirus infection | 6/135 | 226/8846 | 0.1315 | 0.8670 | 0.8670 | TRAF5/HLA-B/GNG4/ADCY1/PDGFRA/CALML4 | 6 |
| hsa00380 | Tryptophan metabolism | 2/135 | 42/8846 | 0.1343 | 0.8670 | 0.8670 | ACAT2/IL4I1 | 2 |
| hsa04975 | Fat digestion and absorption | 2/135 | 43/8846 | 0.1395 | 0.8670 | 0.8670 | ACAT2/PLA2G2C | 2 |
| hsa04512 | ECM-receptor interaction | 3/135 | 89/8846 | 0.1545 | 0.8670 | 0.8670 | COMP/LAMA2/SPP1 | 3 |
| hsa00620 | Pyruvate metabolism | 2/135 | 47/8846 | 0.1608 | 0.8670 | 0.8670 | ACAT2/ACSS1 | 2 |
| hsa05322 | Systemic lupus erythematosus | 4/135 | 139/8846 | 0.1627 | 0.8670 | 0.8670 | H2AC16/H4C11/H2AC20/H2AC4 | 4 |
| hsa04540 | Gap junction | 3/135 | 92/8846 | 0.1656 | 0.8670 | 0.8670 | TUBA1A/ADCY1/PDGFRA | 3 |
| hsa04912 | GnRH signaling pathway | 3/135 | 93/8846 | 0.1693 | 0.8670 | 0.8670 | MMP2/ADCY1/CALML4 | 3 |
| hsa03450 | Non-homologous end-joining | 1/135 | 13/8846 | 0.1813 | 0.8670 | 0.8670 | XRCC4 | 1 |
| hsa04913 | Ovarian steroidogenesis | 2/135 | 51/8846 | 0.1826 | 0.8670 | 0.8670 | STAR/ADCY1 | 2 |
| hsa04979 | Cholesterol metabolism | 2/135 | 51/8846 | 0.1826 | 0.8670 | 0.8670 | STAR/ANGPTL4 | 2 |
| hsa04713 | Circadian entrainment | 3/135 | 97/8846 | 0.1845 | 0.8670 | 0.8670 | GNG4/ADCY1/CALML4 | 3 |
| hsa04970 | Salivary secretion | 3/135 | 97/8846 | 0.1845 | 0.8670 | 0.8670 | ATP2B3/ADCY1/CALML4 | 3 |
| hsa00270 | Cysteine and methionine metabolism | 2/135 | 52/8846 | 0.1881 | 0.8670 | 0.8670 | BCAT1/IL4I1 | 2 |
| hsa04020 | Calcium signaling pathway | 6/135 | 254/8846 | 0.1920 | 0.8670 | 0.8670 | FLT4/P2RX1/ATP2B3/ADCY1/PDGFRA/CALML4 | 6 |
| hsa01522 | Endocrine resistance | 3/135 | 99/8846 | 0.1922 | 0.8670 | 0.8670 | MMP2/NOTCH3/ADCY1 | 3 |
| hsa04750 | Inflammatory mediator regulation of TRP channels | 3/135 | 99/8846 | 0.1922 | 0.8670 | 0.8670 | ASIC1/ADCY1/CALML4 | 3 |
| hsa04961 | Endocrine and other factor-regulated calcium reabsorption | 2/135 | 53/8846 | 0.1936 | 0.8670 | 0.8670 | DNM1/ATP2B3 | 2 |
| hsa04916 | Melanogenesis | 3/135 | 101/8846 | 0.2000 | 0.8670 | 0.8670 | WNT8B/ADCY1/CALML4 | 3 |
| hsa00564 | Glycerophospholipid metabolism | 3/135 | 103/8846 | 0.2079 | 0.8670 | 0.8670 | PLAAT3/PHOSPHO1/PLA2G2C | 3 |
| hsa05414 | Dilated cardiomyopathy | 3/135 | 105/8846 | 0.2158 | 0.8670 | 0.8670 | SGCA/LAMA2/ADCY1 | 3 |
| hsa04218 | Cellular senescence | 4/135 | 157/8846 | 0.2183 | 0.8670 | 0.8670 | RASSF5/HLA-B/IGFBP3/CALML4 | 4 |
| hsa00360 | Phenylalanine metabolism | 1/135 | 16/8846 | 0.2183 | 0.8670 | 0.8670 | IL4I1 | 1 |
| hsa00430 | Taurine and hypotaurine metabolism | 1/135 | 16/8846 | 0.2183 | 0.8670 | 0.8670 | GGT6 | 1 |
| hsa04972 | Pancreatic secretion | 3/135 | 106/8846 | 0.2198 | 0.8670 | 0.8670 | PLA2G2C/ATP2B3/ADCY1 | 3 |
| hsa04217 | Necroptosis | 4/135 | 159/8846 | 0.2248 | 0.8670 | 0.8670 | H2AC16/TRAF5/H2AC20/H2AC4 | 4 |
| hsa04923 | Regulation of lipolysis in adipocytes | 2/135 | 59/8846 | 0.2272 | 0.8670 | 0.8670 | PLAAT3/ADCY1 | 2 |
| hsa04350 | TGF-beta signaling pathway | 3/135 | 108/8846 | 0.2279 | 0.8670 | 0.8670 | FMOD/FBN1/NEO1 | 3 |
| hsa00910 | Nitrogen metabolism | 1/135 | 17/8846 | 0.2302 | 0.8670 | 0.8670 | CA12 | 1 |
| hsa00590 | Arachidonic acid metabolism | 2/135 | 61/8846 | 0.2385 | 0.8844 | 0.8844 | PLAAT3/PLA2G2C | 2 |
| hsa05165 | Human papillomavirus infection | 7/135 | 333/8846 | 0.2461 | 0.8987 | 0.8987 | COMP/NOTCH3/WNT8B/ATP6V0A4/HLA-B/LAMA2/SPP1 | 7 |
| hsa04927 | Cortisol synthesis and secretion | 2/135 | 65/8846 | 0.2612 | 0.9192 | 0.9192 | STAR/ADCY1 | 2 |
| hsa04024 | cAMP signaling pathway | 5/135 | 226/8846 | 0.2628 | 0.9192 | 0.9192 | SSTR1/CNGB1/ATP2B3/ADCY1/CALML4 | 5 |
| hsa00785 | Lipoic acid metabolism | 1/135 | 20/8846 | 0.2650 | 0.9192 | 0.9192 | OGDHL | 1 |
| hsa00010 | Glycolysis / Gluconeogenesis | 2/135 | 67/8846 | 0.2726 | 0.9192 | 0.9192 | ACSS1/ALDOB | 2 |
| hsa04720 | Long-term potentiation | 2/135 | 67/8846 | 0.2726 | 0.9192 | 0.9192 | ADCY1/CALML4 | 2 |
| hsa00770 | Pantothenate and CoA biosynthesis | 1/135 | 21/8846 | 0.2763 | 0.9192 | 0.9192 | BCAT1 | 1 |
| hsa05120 | Epithelial cell signaling in Helicobacter pylori infection | 2/135 | 71/8846 | 0.2953 | 0.9192 | 0.9192 | JAM3/ATP6V0A4 | 2 |
| hsa00220 | Arginine biosynthesis | 1/135 | 23/8846 | 0.2982 | 0.9192 | 0.9192 | GPT | 1 |
| hsa04611 | Platelet activation | 3/135 | 126/8846 | 0.3021 | 0.9192 | 0.9192 | FYN/P2RX1/ADCY1 | 3 |
| hsa00534 | Glycosaminoglycan biosynthesis - heparan sulfate / heparin | 1/135 | 24/8846 | 0.3090 | 0.9192 | 0.9192 | EXTL1 | 1 |
| hsa04360 | Axon guidance | 4/135 | 184/8846 | 0.3090 | 0.9192 | 0.9192 | NRP1/FYN/UNC5B/NEO1 | 4 |
| hsa04926 | Relaxin signaling pathway | 3/135 | 130/8846 | 0.3189 | 0.9192 | 0.9192 | MMP2/GNG4/ADCY1 | 3 |
| hsa03320 | PPAR signaling pathway | 2/135 | 76/8846 | 0.3235 | 0.9192 | 0.9192 | HMGCS1/ANGPTL4 | 2 |
| hsa04971 | Gastric acid secretion | 2/135 | 76/8846 | 0.3235 | 0.9192 | 0.9192 | ADCY1/CALML4 | 2 |
| hsa05214 | Glioma | 2/135 | 76/8846 | 0.3235 | 0.9192 | 0.9192 | PDGFRA/CALML4 | 2 |
| hsa05100 | Bacterial invasion of epithelial cells | 2/135 | 78/8846 | 0.3347 | 0.9192 | 0.9192 | DNM1/SEPTIN6 | 2 |
| hsa04270 | Vascular smooth muscle contraction | 3/135 | 134/8846 | 0.3356 | 0.9192 | 0.9192 | PLA2G2C/ADCY1/CALML4 | 3 |
| hsa04650 | Natural killer cell mediated cytotoxicity | 3/135 | 135/8846 | 0.3398 | 0.9192 | 0.9192 | FYN/ULBP3/HLA-B | 3 |
| hsa04721 | Synaptic vesicle cycle | 2/135 | 79/8846 | 0.3403 | 0.9192 | 0.9192 | DNM1/ATP6V0A4 | 2 |
| hsa04144 | Endocytosis | 5/135 | 252/8846 | 0.3403 | 0.9192 | 0.9192 | ARAP2/DNM1/RAB31/HLA-B/PDGFRA | 5 |
| hsa05167 | Kaposi sarcoma-associated herpesvirus infection | 4/135 | 196/8846 | 0.3507 | 0.9192 | 0.9192 | PREX1/HLA-B/GNG4/CALML4 | 4 |
| hsa00983 | Drug metabolism - other enzymes | 2/135 | 81/8846 | 0.3514 | 0.9192 | 0.9192 | CES1/MPO | 2 |
| hsa04915 | Estrogen signaling pathway | 3/135 | 139/8846 | 0.3566 | 0.9192 | 0.9192 | MMP2/ADCY1/CALML4 | 3 |
| hsa00020 | Citrate cycle (TCA cycle) | 1/135 | 30/8846 | 0.3701 | 0.9192 | 0.9192 | OGDHL | 1 |
| hsa04550 | Signaling pathways regulating pluripotency of stem cells | 3/135 | 144/8846 | 0.3774 | 0.9192 | 0.9192 | WNT8B/HOXA1/ESRRB | 3 |
| hsa05412 | Arrhythmogenic right ventricular cardiomyopathy | 2/135 | 86/8846 | 0.3789 | 0.9192 | 0.9192 | SGCA/LAMA2 | 2 |
| hsa00030 | Pentose phosphate pathway | 1/135 | 31/8846 | 0.3797 | 0.9192 | 0.9192 | ALDOB | 1 |
| hsa00640 | Propanoate metabolism | 1/135 | 32/8846 | 0.3892 | 0.9192 | 0.9192 | ACSS1 | 1 |
| hsa05224 | Breast cancer | 3/135 | 148/8846 | 0.3940 | 0.9192 | 0.9192 | FLT4/NOTCH3/WNT8B | 3 |
| hsa04727 | GABAergic synapse | 2/135 | 89/8846 | 0.3952 | 0.9192 | 0.9192 | GNG4/ADCY1 | 2 |
| hsa04211 | Longevity regulating pathway | 2/135 | 90/8846 | 0.4006 | 0.9192 | 0.9192 | PPARGC1A/ADCY1 | 2 |
| hsa05032 | Morphine addiction | 2/135 | 91/8846 | 0.4060 | 0.9192 | 0.9192 | GNG4/ADCY1 | 2 |
| hsa00051 | Fructose and mannose metabolism | 1/135 | 34/8846 | 0.4078 | 0.9192 | 0.9192 | ALDOB | 1 |
| hsa05170 | Human immunodeficiency virus 1 infection | 4/135 | 213/8846 | 0.4098 | 0.9192 | 0.9192 | TRAF5/HLA-B/GNG4/CALML4 | 4 |
| hsa04520 | Adherens junction | 2/135 | 93/8846 | 0.4167 | 0.9192 | 0.9192 | FYN/PTPRJ | 2 |
| hsa05222 | Small cell lung cancer | 2/135 | 93/8846 | 0.4167 | 0.9192 | 0.9192 | TRAF5/LAMA2 | 2 |
| hsa04261 | Adrenergic signaling in cardiomyocytes | 3/135 | 154/8846 | 0.4187 | 0.9192 | 0.9192 | ATP2B3/ADCY1/CALML4 | 3 |
| hsa04921 | Oxytocin signaling pathway | 3/135 | 154/8846 | 0.4187 | 0.9192 | 0.9192 | KCNJ12/ADCY1/CALML4 | 3 |
| hsa04934 | Cushing syndrome | 3/135 | 155/8846 | 0.4228 | 0.9192 | 0.9192 | WNT8B/STAR/ADCY1 | 3 |
| hsa00350 | Tyrosine metabolism | 1/135 | 36/8846 | 0.4258 | 0.9192 | 0.9192 | IL4I1 | 1 |
| hsa00512 | Mucin type O-glycan biosynthesis | 1/135 | 36/8846 | 0.4258 | 0.9192 | 0.9192 | GALNT16 | 1 |
| hsa05143 | African trypanosomiasis | 1/135 | 37/8846 | 0.4346 | 0.9192 | 0.9192 | APOL1 | 1 |
| hsa05330 | Allograft rejection | 1/135 | 38/8846 | 0.4432 | 0.9192 | 0.9192 | HLA-B | 1 |
| hsa05231 | Choline metabolism in cancer | 2/135 | 99/8846 | 0.4480 | 0.9192 | 0.9192 | SLC44A2/PDGFRA | 2 |
| hsa05410 | Hypertrophic cardiomyopathy | 2/135 | 99/8846 | 0.4480 | 0.9192 | 0.9192 | SGCA/LAMA2 | 2 |
| hsa05146 | Amoebiasis | 2/135 | 103/8846 | 0.4685 | 0.9192 | 0.9192 | LAMA2/ADCY1 | 2 |
| hsa05219 | Bladder cancer | 1/135 | 41/8846 | 0.4685 | 0.9192 | 0.9192 | MMP2 | 1 |
| hsa00513 | Various types of N-glycan biosynthesis | 1/135 | 42/8846 | 0.4766 | 0.9192 | 0.9192 | TUSC3 | 1 |
| hsa04216 | Ferroptosis | 1/135 | 42/8846 | 0.4766 | 0.9192 | 0.9192 | SLC7A11 | 1 |
| hsa04151 | PI3K-Akt signaling pathway | 6/135 | 362/8846 | 0.4778 | 0.9192 | 0.9192 | FLT4/COMP/LAMA2/SPP1/GNG4/PDGFRA | 6 |
| hsa04974 | Protein digestion and absorption | 2/135 | 105/8846 | 0.4785 | 0.9192 | 0.9192 | SLC16A10/COL8A2 | 2 |
| hsa04530 | Tight junction | 3/135 | 170/8846 | 0.4827 | 0.9192 | 0.9192 | CLDN2/JAM3/TUBA1A | 3 |
| hsa00071 | Fatty acid degradation | 1/135 | 43/8846 | 0.4846 | 0.9192 | 0.9192 | ACAT2 | 1 |
| hsa04940 | Type I diabetes mellitus | 1/135 | 43/8846 | 0.4846 | 0.9192 | 0.9192 | HLA-B | 1 |
| hsa04010 | MAPK signaling pathway | 5/135 | 300/8846 | 0.4854 | 0.9192 | 0.9192 | MAPT/FLT4/DUSP4/MAP3K20/PDGFRA | 5 |
| hsa04922 | Glucagon signaling pathway | 2/135 | 107/8846 | 0.4884 | 0.9192 | 0.9192 | PPARGC1A/CALML4 | 2 |
| hsa05332 | Graft-versus-host disease | 1/135 | 44/8846 | 0.4925 | 0.9192 | 0.9192 | HLA-B | 1 |
| hsa02010 | ABC transporters | 1/135 | 45/8846 | 0.5003 | 0.9192 | 0.9192 | ABCA3 | 1 |
| hsa00514 | Other types of O-glycan biosynthesis | 1/135 | 47/8846 | 0.5155 | 0.9192 | 0.9192 | GALNT16 | 1 |
| hsa05152 | Tuberculosis | 3/135 | 180/8846 | 0.5210 | 0.9192 | 0.9192 | ATP6V0A4/ITGAX/CALML4 | 3 |
| hsa04928 | Parathyroid hormone synthesis, secretion and action | 2/135 | 115/8846 | 0.5269 | 0.9192 | 0.9192 | MMP24/ADCY1 | 2 |
| hsa05030 | Cocaine addiction | 1/135 | 49/8846 | 0.5303 | 0.9192 | 0.9192 | GRM2 | 1 |
| hsa00330 | Arginine and proline metabolism | 1/135 | 50/8846 | 0.5375 | 0.9192 | 0.9192 | PRODH | 1 |
| hsa05144 | Malaria | 1/135 | 50/8846 | 0.5375 | 0.9192 | 0.9192 | COMP | 1 |
| hsa05110 | Vibrio cholerae infection | 1/135 | 51/8846 | 0.5446 | 0.9192 | 0.9192 | ATP6V0A4 | 1 |
| hsa05010 | Alzheimer disease | 6/135 | 391/8846 | 0.5537 | 0.9192 | 0.9192 | MAPT/TUBA1A/COX4I2/WNT8B/APBB1/CALML4 | 6 |
| hsa00510 | N-Glycan biosynthesis | 1/135 | 53/8846 | 0.5585 | 0.9192 | 0.9192 | TUSC3 | 1 |
| hsa05320 | Autoimmune thyroid disease | 1/135 | 53/8846 | 0.5585 | 0.9192 | 0.9192 | HLA-B | 1 |
| hsa04071 | Sphingolipid signaling pathway | 2/135 | 122/8846 | 0.5589 | 0.9192 | 0.9192 | FYN/OPRD1 | 2 |
| hsa04152 | AMPK signaling pathway | 2/135 | 122/8846 | 0.5589 | 0.9192 | 0.9192 | CAB39L/PPARGC1A | 2 |
| hsa00600 | Sphingolipid metabolism | 1/135 | 54/8846 | 0.5652 | 0.9192 | 0.9192 | B4GALT6 | 1 |
| hsa04062 | Chemokine signaling pathway | 3/135 | 193/8846 | 0.5685 | 0.9192 | 0.9192 | PREX1/GNG4/ADCY1 | 3 |
| hsa05202 | Transcriptional misregulation in cancer | 3/135 | 193/8846 | 0.5685 | 0.9192 | 0.9192 | MPO/ARNT2/IGFBP3 | 3 |
| hsa03460 | Fanconi anemia pathway | 1/135 | 55/8846 | 0.5719 | 0.9192 | 0.9192 | SLX1B | 1 |
| hsa05134 | Legionellosis | 1/135 | 56/8846 | 0.5785 | 0.9192 | 0.9192 | EEF1A2 | 1 |
| hsa01212 | Fatty acid metabolism | 1/135 | 57/8846 | 0.5850 | 0.9192 | 0.9192 | ACAT2 | 1 |
| hsa00480 | Glutathione metabolism | 1/135 | 58/8846 | 0.5913 | 0.9192 | 0.9192 | GGT6 | 1 |
| hsa05012 | Parkinson disease | 4/135 | 271/8846 | 0.5976 | 0.9192 | 0.9192 | MAPT/TUBA1A/COX4I2/CALML4 | 4 |
| hsa04142 | Lysosome | 2/135 | 132/8846 | 0.6020 | 0.9192 | 0.9192 | ATP6V0A4/DMXL2 | 2 |
| hsa04728 | Dopaminergic synapse | 2/135 | 132/8846 | 0.6020 | 0.9192 | 0.9192 | GNG4/CALML4 | 2 |
| hsa05130 | Pathogenic Escherichia coli infection | 3/135 | 203/8846 | 0.6030 | 0.9192 | 0.9192 | CLDN2/FYN/TUBA1A | 3 |
| hsa05169 | Epstein-Barr virus infection | 3/135 | 203/8846 | 0.6030 | 0.9192 | 0.9192 | TRAF5/HLA-B/VIM | 3 |
| hsa05205 | Proteoglycans in cancer | 3/135 | 204/8846 | 0.6063 | 0.9192 | 0.9192 | MMP2/TIMP3/WNT8B | 3 |
| hsa05203 | Viral carcinogenesis | 3/135 | 205/8846 | 0.6097 | 0.9192 | 0.9192 | TRAF5/H4C11/HLA-B | 3 |
| hsa04213 | Longevity regulating pathway - multiple species | 1/135 | 62/8846 | 0.6159 | 0.9192 | 0.9192 | ADCY1 | 1 |
| hsa04330 | Notch signaling pathway | 1/135 | 62/8846 | 0.6159 | 0.9192 | 0.9192 | NOTCH3 | 1 |
| hsa00310 | Lysine degradation | 1/135 | 63/8846 | 0.6218 | 0.9192 | 0.9192 | ACAT2 | 1 |
| hsa05217 | Basal cell carcinoma | 1/135 | 63/8846 | 0.6218 | 0.9192 | 0.9192 | WNT8B | 1 |
| hsa00190 | Oxidative phosphorylation | 2/135 | 138/8846 | 0.6263 | 0.9192 | 0.9192 | COX4I2/ATP6V0A4 | 2 |
| hsa04910 | Insulin signaling pathway | 2/135 | 138/8846 | 0.6263 | 0.9192 | 0.9192 | PPARGC1A/CALML4 | 2 |
| hsa03250 | Viral life cycle - HIV-1 | 1/135 | 64/8846 | 0.6276 | 0.9192 | 0.9192 | MAP1B | 1 |
| hsa04114 | Oocyte meiosis | 2/135 | 139/8846 | 0.6303 | 0.9192 | 0.9192 | ADCY1/CALML4 | 2 |
| hsa04929 | GnRH secretion | 1/135 | 65/8846 | 0.6333 | 0.9192 | 0.9192 | SPP1 | 1 |
| hsa05418 | Fluid shear stress and atherosclerosis | 2/135 | 141/8846 | 0.6380 | 0.9192 | 0.9192 | MMP2/CALML4 | 2 |
| hsa00830 | Retinol metabolism | 1/135 | 68/8846 | 0.6500 | 0.9192 | 0.9192 | PNPLA4 | 1 |
| hsa05221 | Acute myeloid leukemia | 1/135 | 68/8846 | 0.6500 | 0.9192 | 0.9192 | MPO | 1 |
| hsa04664 | Fc epsilon RI signaling pathway | 1/135 | 69/8846 | 0.6554 | 0.9192 | 0.9192 | FYN | 1 |
| hsa04924 | Renin secretion | 1/135 | 69/8846 | 0.6554 | 0.9192 | 0.9192 | CALML4 | 1 |
| hsa05031 | Amphetamine addiction | 1/135 | 69/8846 | 0.6554 | 0.9192 | 0.9192 | CALML4 | 1 |
| hsa04920 | Adipocytokine signaling pathway | 1/135 | 70/8846 | 0.6607 | 0.9192 | 0.9192 | PPARGC1A | 1 |
| hsa05211 | Renal cell carcinoma | 1/135 | 70/8846 | 0.6607 | 0.9192 | 0.9192 | ARNT2 | 1 |
| hsa05230 | Central carbon metabolism in cancer | 1/135 | 71/8846 | 0.6659 | 0.9192 | 0.9192 | PDGFRA | 1 |
| hsa05166 | Human T-cell leukemia virus 1 infection | 3/135 | 223/8846 | 0.6664 | 0.9192 | 0.9192 | NRP1/HLA-B/ADCY1 | 3 |
| hsa04723 | Retrograde endocannabinoid signaling | 2/135 | 149/8846 | 0.6679 | 0.9192 | 0.9192 | GNG4/ADCY1 | 2 |
| hsa05208 | Chemical carcinogenesis - reactive oxygen species | 3/135 | 226/8846 | 0.6753 | 0.9192 | 0.9192 | COX4I2/PTPRJ/PRKD1 | 3 |
| hsa00562 | Inositol phosphate metabolism | 1/135 | 73/8846 | 0.6761 | 0.9192 | 0.9192 | PLCH2 | 1 |
| hsa05218 | Melanoma | 1/135 | 73/8846 | 0.6761 | 0.9192 | 0.9192 | PDGFRA | 1 |
| hsa05223 | Non-small cell lung cancer | 1/135 | 73/8846 | 0.6761 | 0.9192 | 0.9192 | RASSF5 | 1 |
| hsa04115 | p53 signaling pathway | 1/135 | 75/8846 | 0.6860 | 0.9192 | 0.9192 | IGFBP3 | 1 |
| hsa04918 | Thyroid hormone synthesis | 1/135 | 75/8846 | 0.6860 | 0.9192 | 0.9192 | ADCY1 | 1 |
| hsa04810 | Regulation of actin cytoskeleton | 3/135 | 230/8846 | 0.6868 | 0.9192 | 0.9192 | ITGAX/FGD1/PDGFRA | 3 |
| hsa05140 | Leishmaniasis | 1/135 | 77/8846 | 0.6956 | 0.9192 | 0.9192 | EEF1A2 | 1 |
| hsa04148 | Efferocytosis | 2/135 | 157/8846 | 0.6957 | 0.9192 | 0.9192 | DUSP4/ANO5 | 2 |
| hsa04390 | Hippo signaling pathway | 2/135 | 157/8846 | 0.6957 | 0.9192 | 0.9192 | WNT8B/NKD2 | 2 |
| hsa04150 | mTOR signaling pathway | 2/135 | 158/8846 | 0.6991 | 0.9192 | 0.9192 | CAB39L/WNT8B | 2 |
| hsa05133 | Pertussis | 1/135 | 78/8846 | 0.7003 | 0.9192 | 0.9192 | CALML4 | 1 |
| hsa04714 | Thermogenesis | 3/135 | 235/8846 | 0.7008 | 0.9192 | 0.9192 | COX4I2/PPARGC1A/ADCY1 | 3 |
| hsa05206 | MicroRNAs in cancer | 4/135 | 312/8846 | 0.7068 | 0.9192 | 0.9192 | TIMP3/NOTCH3/VIM/PDGFRA | 4 |
| hsa01521 | EGFR tyrosine kinase inhibitor resistance | 1/135 | 80/8846 | 0.7094 | 0.9192 | 0.9192 | PDGFRA | 1 |
| hsa04612 | Antigen processing and presentation | 1/135 | 80/8846 | 0.7094 | 0.9192 | 0.9192 | HLA-B | 1 |
| hsa03083 | Polycomb repressive complex | 1/135 | 83/8846 | 0.7226 | 0.9264 | 0.9264 | YY1 | 1 |
| hsa04146 | Peroxisome | 1/135 | 83/8846 | 0.7226 | 0.9264 | 0.9264 | MVK | 1 |
| hsa04911 | Insulin secretion | 1/135 | 86/8846 | 0.7353 | 0.9284 | 0.9284 | ADCY1 | 1 |
| hsa04141 | Protein processing in endoplasmic reticulum | 2/135 | 170/8846 | 0.7369 | 0.9284 | 0.9284 | TUSC3/DNAJC5G | 2 |
| hsa04260 | Cardiac muscle contraction | 1/135 | 87/8846 | 0.7393 | 0.9284 | 0.9284 | COX4I2 | 1 |
| hsa04610 | Complement and coagulation cascades | 1/135 | 88/8846 | 0.7434 | 0.9284 | 0.9284 | ITGAX | 1 |
| hsa04310 | Wnt signaling pathway | 2/135 | 174/8846 | 0.7486 | 0.9284 | 0.9284 | WNT8B/NKD2 | 2 |
| hsa04976 | Bile secretion | 1/135 | 90/8846 | 0.7512 | 0.9284 | 0.9284 | ADCY1 | 1 |
| hsa05022 | Pathways of neurodegeneration - multiple diseases | 6/135 | 483/8846 | 0.7538 | 0.9284 | 0.9284 | MAPT/TUBA1A/COX4I2/WNT8B/RAB39B/CALML4 | 6 |
| hsa04658 | Th1 and Th2 cell differentiation | 1/135 | 92/8846 | 0.7588 | 0.9284 | 0.9284 | NOTCH3 | 1 |
| hsa05323 | Rheumatoid arthritis | 1/135 | 94/8846 | 0.7662 | 0.9284 | 0.9284 | ATP6V0A4 | 1 |
| hsa04657 | IL-17 signaling pathway | 1/135 | 95/8846 | 0.7698 | 0.9284 | 0.9284 | TRAF5 | 1 |
| hsa04070 | Phosphatidylinositol signaling system | 1/135 | 98/8846 | 0.7803 | 0.9284 | 0.9284 | CALML4 | 1 |
| hsa05215 | Prostate cancer | 1/135 | 98/8846 | 0.7803 | 0.9284 | 0.9284 | PDGFRA | 1 |
| hsa04933 | AGE-RAGE signaling pathway in diabetic complications | 1/135 | 101/8846 | 0.7903 | 0.9284 | 0.9284 | MMP2 | 1 |
| hsa03015 | mRNA surveillance pathway | 1/135 | 103/8846 | 0.7967 | 0.9284 | 0.9284 | MSI1 | 1 |
| hsa05142 | Chagas disease | 1/135 | 103/8846 | 0.7967 | 0.9284 | 0.9284 | ADCY1 | 1 |
| hsa05020 | Prion disease | 3/135 | 278/8846 | 0.8022 | 0.9284 | 0.9284 | FYN/TUBA1A/COX4I2 | 3 |
| hsa04064 | NF-kappa B signaling pathway | 1/135 | 105/8846 | 0.8030 | 0.9284 | 0.9284 | TRAF5 | 1 |
| hsa04625 | C-type lectin receptor signaling pathway | 1/135 | 105/8846 | 0.8030 | 0.9284 | 0.9284 | CALML4 | 1 |
| hsa03013 | Nucleocytoplasmic transport | 1/135 | 108/8846 | 0.8120 | 0.9284 | 0.9284 | EEF1A2 | 1 |
| hsa04066 | HIF-1 signaling pathway | 1/135 | 109/8846 | 0.8149 | 0.9284 | 0.9284 | ALDOB | 1 |
| hsa04620 | Toll-like receptor signaling pathway | 1/135 | 109/8846 | 0.8149 | 0.9284 | 0.9284 | SPP1 | 1 |
| hsa04931 | Insulin resistance | 1/135 | 109/8846 | 0.8149 | 0.9284 | 0.9284 | PPARGC1A | 1 |
| hsa04080 | Neuroactive ligand-receptor interaction | 4/135 | 368/8846 | 0.8192 | 0.9284 | 0.9284 | SSTR1/P2RX1/OPRD1/GRM2 | 4 |
| hsa04914 | Progesterone-mediated oocyte maturation | 1/135 | 111/8846 | 0.8205 | 0.9284 | 0.9284 | ADCY1 | 1 |
| hsa05145 | Toxoplasmosis | 1/135 | 111/8846 | 0.8205 | 0.9284 | 0.9284 | LAMA2 | 1 |
| hsa05415 | Diabetic cardiomyopathy | 2/135 | 205/8846 | 0.8249 | 0.9289 | 0.9289 | MMP2/COX4I2 | 2 |
| hsa04726 | Serotonergic synapse | 1/135 | 115/8846 | 0.8314 | 0.9319 | 0.9319 | GNG4 | 1 |
| hsa04668 | TNF signaling pathway | 1/135 | 119/8846 | 0.8416 | 0.9338 | 0.9338 | TRAF5 | 1 |
| hsa04722 | Neurotrophin signaling pathway | 1/135 | 120/8846 | 0.8440 | 0.9338 | 0.9338 | CALML4 | 1 |
| hsa05207 | Chemical carcinogenesis - receptor activation | 2/135 | 215/8846 | 0.8447 | 0.9338 | 0.9338 | PAQR8/ADCY1 | 2 |
| hsa04660 | T cell receptor signaling pathway | 1/135 | 122/8846 | 0.8488 | 0.9341 | 0.9341 | FYN | 1 |
| hsa05016 | Huntington disease | 3/135 | 311/8846 | 0.8591 | 0.9404 | 0.9404 | TUBA1A/COX4I2/PPARGC1A | 3 |
| hsa00230 | Purine metabolism | 1/135 | 128/8846 | 0.8623 | 0.9404 | 0.9404 | ADCY1 | 1 |
| hsa04068 | FoxO signaling pathway | 1/135 | 133/8846 | 0.8727 | 0.9473 | 0.9473 | HOMER2 | 1 |
| hsa04210 | Apoptosis | 1/135 | 136/8846 | 0.8785 | 0.9494 | 0.9494 | TUBA1A | 1 |
| hsa04380 | Osteoclast differentiation | 1/135 | 142/8846 | 0.8894 | 0.9563 | 0.9563 | FYN | 1 |
| hsa04936 | Alcoholic liver disease | 1/135 | 144/8846 | 0.8928 | 0.9563 | 0.9563 | PPARGC1A | 1 |
| hsa05131 | Shigellosis | 2/135 | 249/8846 | 0.8978 | 0.9564 | 0.9564 | TRAF5/SEPTIN6 | 2 |
| hsa05226 | Gastric cancer | 1/135 | 150/8846 | 0.9024 | 0.9564 | 0.9564 | WNT8B | 1 |
| hsa01240 | Biosynthesis of cofactors | 1/135 | 153/8846 | 0.9069 | 0.9564 | 0.9564 | BCAT1 | 1 |
| hsa04932 | Non-alcoholic fatty liver disease | 1/135 | 157/8846 | 0.9125 | 0.9564 | 0.9564 | COX4I2 | 1 |
| hsa05160 | Hepatitis C | 1/135 | 159/8846 | 0.9152 | 0.9564 | 0.9564 | CLDN2 | 1 |
| hsa04630 | JAK-STAT signaling pathway | 1/135 | 168/8846 | 0.9264 | 0.9564 | 0.9564 | PDGFRA | 1 |
| hsa05014 | Amyotrophic lateral sclerosis | 3/135 | 371/8846 | 0.9269 | 0.9564 | 0.9564 | TUBA1A/COX4I2/RAB39B | 3 |
| hsa04140 | Autophagy - animal | 1/135 | 169/8846 | 0.9275 | 0.9564 | 0.9564 | RAB39B | 1 |
| hsa05225 | Hepatocellular carcinoma | 1/135 | 170/8846 | 0.9286 | 0.9564 | 0.9564 | WNT8B | 1 |
| hsa04621 | NOD-like receptor signaling pathway | 1/135 | 189/8846 | 0.9470 | 0.9712 | 0.9712 | TRAF5 | 1 |
| hsa04814 | Motor proteins | 1/135 | 197/8846 | 0.9533 | 0.9735 | 0.9735 | TUBA1A | 1 |
| hsa05417 | Lipid and atherosclerosis | 1/135 | 216/8846 | 0.9654 | 0.9817 | 0.9817 | CALML4 | 1 |
| hsa04740 | Olfactory transduction | 3/135 | 453/8846 | 0.9722 | 0.9837 | 0.9837 | OR1F1/CNGB1/CALML4 | 3 |
| hsa05171 | Coronavirus disease - COVID-19 | 1/135 | 238/8846 | 0.9755 | 0.9837 | 0.9837 | NRP1 | 1 |
| hsa05132 | Salmonella infection | 1/135 | 251/8846 | 0.9801 | 0.9842 | 0.9842 | TUBA1A | 1 |
| hsa04060 | Cytokine-cytokine receptor interaction | 1/135 | 298/8846 | 0.9906 | 0.9906 | 0.9906 | GDF11 | 1 |
